# Interactive Roles of Metabolic Dysfunction‐Associated Steatotic Liver Disease and Nutritional Status in the Prognosis of Patients With Hepatocellular Carcinoma After Surgical Resection

**DOI:** 10.1002/kjm2.70256

**Published:** 2026-07-03

**Authors:** Chun‐Ting Ho, Chien‐An Liu, Chia‐Jung Ho, Yi‐Chen Lin, Shin‐Yu Tsai, Hao‐Jan Lei, Shu‐Cheng Chou, Yi‐Hsiang Huang, Ming‐Chih Hou, Jaw‐Ching Wu, Chien‐Wei Su

**Affiliations:** ^1^ Division of General Medicine, Department of Medicine Taipei Veterans General Hospital Taipei Taiwan; ^2^ Ph.D. Program of Interdisciplinary Medicine, College of Medicine National Yang Ming Chiao Tung University Taipei Taiwan; ^3^ Department of Radiology Taipei Veterans General Hospital Taipei Taiwan; ^4^ School of Medicine, College of Medicine National Yang Ming Chiao Tung University Taipei Taiwan; ^5^ Division of General Surgery, Department of Surgery Taipei Veterans General Hospital Taipei Taiwan; ^6^ Division of Gastroenterology and Hepatology, Department of Medicine Taipei Veterans General Hospital Taipei Taiwan; ^7^ Department of Medical Research Taipei Veterans General Hospital Taipei Taiwan; ^8^ Institute of Clinical Medicine, School of Medicine National Yang Ming Chiao Tung University Taipei Taiwan

**Keywords:** hepatocellular carcinoma (HCC), inflammation, metabolic dysfunction‐associated steatotic liver disease (MASLD), nutrition, prognosis

## Abstract

The prognostic effect of concurrent metabolic dysfunction‐associated steatotic liver disease (MASLD) on outcomes for hepatocellular carcinoma (HCC) patients remains unclear. The Prognostic Nutritional Index (PNI) reflects nutritional and inflammatory status and is a well‐known prognostic factor. We therefore aimed to evaluate and compare the prognostic role of PNI in patients with HCC with and without concurrent MASLD undergoing curative surgical resection. In our retrospective study, 991 patients undergoing curative HCC resection were stratified according to MASLD status and pre‐operative PNI (cut‐off: 45). Overall survival (OS) and recurrence‐free survival (RFS) were analyzed using Kaplan–Meier methods and Cox proportional hazards models. Further subgroup analysis was performed according to their concurrent viral hepatitis status. High PNI was identified as an independent protective factor against poor OS for the entire cohort (hazard ratio [HR]: 0.65; 95% confidence interval [CI]: 0.49–0.87, *p* = 0.004). The Kaplan–Meier analysis showed that the high‐PNI/MASLD group had the best OS, while the low‐PNI/no‐MASLD group had the worst. Subgroup analysis indicated that high PNI was an independent protective factor for OS only in the no‐MASLD group (HR: 0.59, *p =* 0.003) but not the MASLD group (HR: 0.94, *p =* 0.793). Its prognostic effect was more significant in HCC patients without concurrent MASLD or with chronic hepatitis B. To conclude, a better nutritional status, assessed by PNI, is a valuable prognostic marker for patients with HCC. Its protective effect on OS is significantly pronounced in patients without concurrent MASLD, indicating that MASLD status modifies the prognostic utility of PNI.

## 1 Introduction

Liver cancer has become one of the leading causes of cancer death, with approximately 757,948 deaths occurring annually worldwide [1]. Despite progress in treatment and prevention, the burden of liver cancer is still rising [2]. Hepatocellular carcinoma (HCC) is the main subtype of primary liver cancer and is well recognized for its poor prognosis, with an estimated 5‐year overall survival (OS) rate around 20% [3]. Early diagnosis, risk stratification, and prompt management are key elements for improving outcomes [4].

Metabolic dysfunction‐associated steatotic liver disease (MASLD) is defined by the simultaneous presence of cardiometabolic risk factors (CMRFs) and steatotic change in the liver noted by imaging or histology. The prevalence of MASLD has increased, especially in developed countries [5]. Its estimated overall prevalence is 30%–40% in general populations and is even higher in specific subgroups, such as patients with type 2 diabetes mellitus (DM) [6]. MASLD can be involved in the etiology of HCC and can also co‐exist with other etiologies, such as chronic viral hepatitis [3, 7, 8].

The impact of MASLD on the prognosis of liver disease and patients with HCC is debated. Several studies have indicated MASLD as a risk factor for poor survival, while others have reported more favorable outcomes in patients with MASLD [9, 10, 11]. Therefore, this knowledge gap needs to be addressed.

The Prognostic Nutritional Index (PNI) is a non‐invasive serum biomarker (NISB) that reflects nutritional and inflammatory status and has shown an ability to predict outcomes and stratify patients into different risk groups, including those with HCC [12, 13]. Moreover, nutritional and inflammatory status are important field factors that interfere with survival and can be altered in the presence of MASLD [14, 15]. Therefore, the aim of this study was to evaluate the prognostic role of nutritional status assessed by PNI in patients with HCC and concurrent MASLD who are undergoing curative surgical resection.

## 2 Methods

### 2.1 Data Source and Study Population

This retrospective cohort study used HCC registry data obtained from Taipei Veterans General Hospital, a major medical center in Northern Taiwan. Newly diagnosed patients with HCC were prospectively enrolled in the registry, with comprehensive information that includes demographics, etiology, laboratory results, tumor characteristics, pathological features, treatments, and health outcomes. Data extracted from this registry have been used in several studies [11, 12, 16, 17].

**FIGURE 1:**
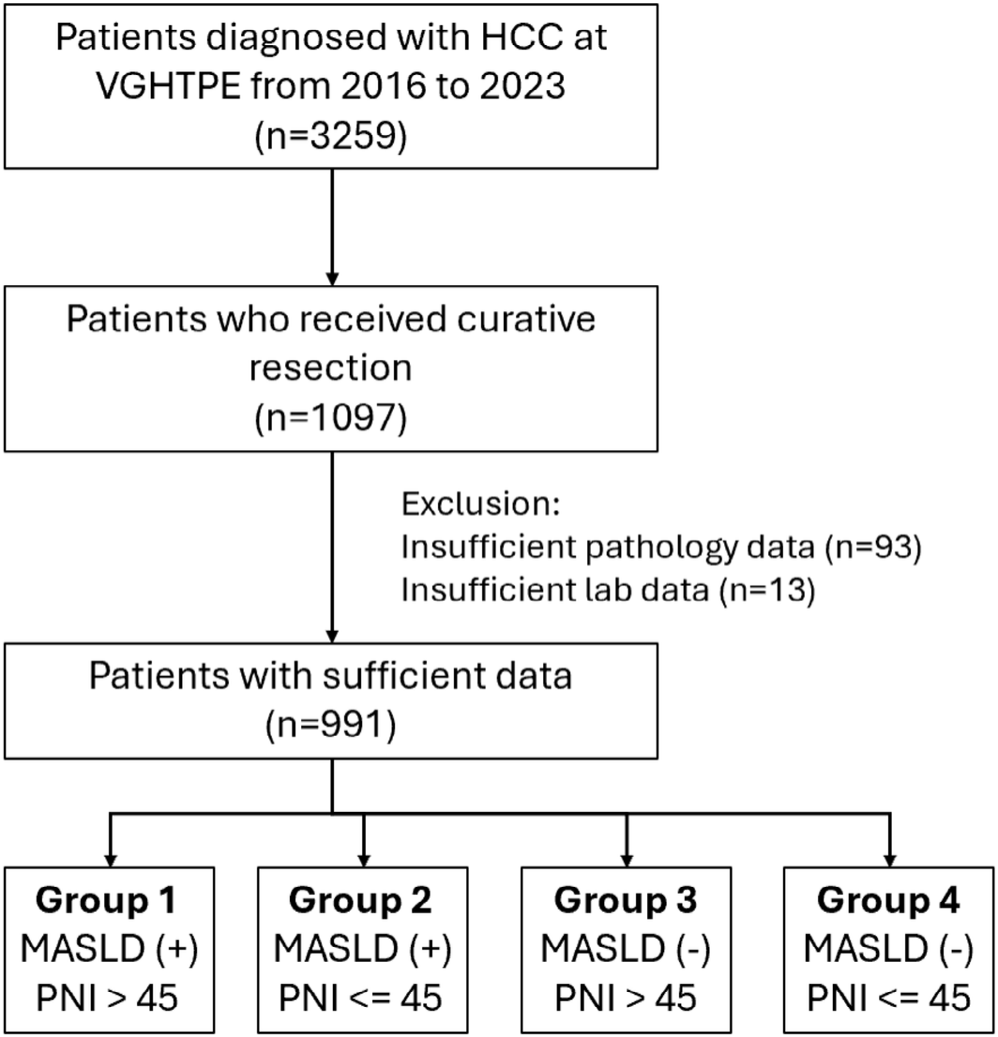
Study flowchart.

The inclusion criteria for curative hepatectomy at our center were as follows: (1) liver function of Child–Pugh grade A or B; (2) tumors involving no more than two Healey segments and no involvement of the main portal vein trunk; (3) absence of extrahepatic tumor dissemination; and (4) absence of other major diseases that could complicate resection [18, 19]. The study retrospectively enrolled treatment‐naïve patients with HCC diagnosed at Taipei Veterans General Hospital between 2016 and 2023 who met these criteria and underwent curative hepatectomy within 3 months after HCC diagnosis. Patients with insufficient clinical, pathological, and radiological data were excluded, resulting in 991 patients eligible for further analysis (Figure 1).

The index date was defined as the date of HCC diagnosis. Patients were followed until death, loss to follow‐up, or the end of the study (30 March 2025). This study adhered to the Declaration of Helsinki and was approved by the Institutional Review Board (IRB) of Taipei Veterans General Hospital (IRB number: 2024‐09‐015CC). The informed consent requirement was waived by the IRB because of the retrospective observational design of the study, and patient information was de‐identified prior to study initiation.

### 2.2 Definitions

Steatotic liver disease (SLD) was identified based on the presence of more than 5% steatosis in the non‐tumor part of operative pathology specimens obtained from curative hepatectomy. The definitions of MASLD and MASH are those of the 2023 nomenclature updated by the American Association for the Study of Liver Diseases (AASLD). MASLD was defined as the coexistence of SLD and at least one CMRF [20]. Patients with MASLD who also exhibited histopathological evidence of steatohepatitis were considered to have metabolic dysfunction‐associated steatohepatitis (MASH).

### 2.3 Patient Stratification and Risk Factor Identification

Patients with HCC were divided into cohorts with and without concurrent MASLD based on the established definition. The two cohorts were divided into subgroups of high and low PNI using a cut‐off of 45. This cut‐off was determined by maximizing the Youden index for survival in accordance with the literature [12]. Using this method, four subgroups of patients were identified: group 1: MASLD and high PNI; group 2: MASLD and low PNI; group 3: no MASLD and high PNI; and group 4: no MASLD and low PNI. Demographic data and survival outcomes were compared among the four subgroups.

### 2.4 Outcome Measurement

The primary outcome of the study was OS, while recurrence‐free survival (RFS) was the secondary outcome. The survival status of each patient was obtained from electronic health records and follow‐up telephone interviews conducted by trained nurses. The index date was defined as the date of HCC diagnosis. Patients were followed until death, loss to follow‐up, or the end of the study period. Additionally, tumor recurrence was classified into two categories: (A) early recurrence, defined as tumor recurrence within 2 years after resection, and (B) late recurrence, defined as tumor recurrence occurring more than 2 years post‐resection [21].

### 2.5 Statistical Analysis

Demographic data, pathological features, tumor‐related factors, viral hepatitis status, and serum biochemistries were collected upon enrolment. Continuous variables are presented as medians with interquartile ranges (IQRs) and were compared using a two‐tailed *t*‐test or Mann–Whitney *U*‐test as appropriate. Categorical variables are expressed as frequencies with percentages and were analyzed using the chi‐squared test or Fisher's exact test.

A Cox proportional hazards model was used to identify significant variables associated with OS and RFS. Variables that reached statistical significance (*p* < 0.05) in the univariate analysis were incorporated into a multivariable Cox model using a backward method. Cumulative OS and RFS rates were estimated using the Kaplan–Meier (KM) method and compared using the log‐rank test. The statistical analyses were performed using R software version 4.4.3 (R Foundation for Statistical Computing, Vienna, Austria). The following R packages were employed for data analysis: Cox modeling, Kaplan–Meier survival analysis, and the plotting packages survival, survminer, caret, pROC, dplyr, and ggplot2.

## 3 Results

### 3.1 Basic Characteristics

Among the 991 patients with HCC who underwent curative hepatectomy, 472 (47.6%) had concurrent MASLD, while the remaining 519 did not. Regarding tumor staging, the cohort included 77 patients in BCLC stage 0, 552 patients in BCLC stage A, 293 patients in BCLC stage B, and 69 patients in BCLC stage C. Among all enrolled patients, 685 (69.1%) had positive serology findings for viral hepatitis B or C. Clinically, over 98% of patients had preserved liver function (Child–Pugh class A), and 86.3% were classified as ALBI grade 1 upon diagnosis.

After stratification using PNI and MASLD status, the four subgroups demonstrated significant differences in baseline characteristics, including age, tumor characteristics (size, stage, number, vascular invasion, and distant metastasis), serum biochemistry profile (platelet, albumin, alanine transaminase [ALT], aspartate transaminase [AST], alkaline phosphatase [ALK‐P], and alpha‐fetoprotein [AFP]), and liver function reserve. Significant differences were also noted in the fibrotic and inflammatory status in the non‐tumor parts of the surgical specimen. Detailed baseline characteristics are presented in Table 1.

**TABLE 1:** Baseline characteristics of the four groups of patients stratified by MASLD and PNI status.

	All (*n* = 991)	Group 1 (*n* = 381)	Group 2 (*n* = 91)	Group 3 (*n* = 360)	Group 4 (*n* = 159)	*p*
Baseline characteristics						
Age (years)	66 (58–73)	65 (57–71)	68 (58–73)	65 (58–71)	69 (61–77)	< 0.001
Sex (M/F)	716/275 (72.3%/27.7%)	283/98 (74.3%/25.7%)	61/30 (67.0%/33.0%)	258/102 (71.7%/28.3%)	114/45 (71.7%/28.3%)	0.551
BCLC stage (0/A/B/C/D)	77/552/293/69/0 (7.8%/55.7%/29.6%/7.0%/0%)	40/235/91/15/0 (10.5%/61.7%/23.0%/3.94%/0%)	6/56/26/3/0 (6.6%/61.5%/28.6%/3.30%/0%)	28/177/122/33/0 (7.8%/49.2%/33.9%/9.2%/0%)	3/84/54/18/0 (1.9%/52.8%/34.0%/11.3%/0%)	< 0.001
Child–Pugh (A/B)	975/16 (98.4%/1.6%)	378/3 (99.2%/0.8%)	88/3 (96.7%/3.3%)	358/2 (99.4%/0.6%)	153/3 (96.2%/3.8%)	0.008
Extrahepatic metastasis (yes/no)	16/975 (1.6%/98.4%)	2/379 (0.5%/99.5%)	0/91 (0%/100%)	8/352 (2.2%/97.8%)	6/153 (3.8%/96.2%)	0.019
Tumor number (single/multiple)	677/314 (68.3%/31.7%)	296/85 (77.7%/22.3%)	59/32 (64.8%/35.2%)	232/128 (64.4%/35.6%)	90/69 (56.6%/43.4%)	< 0.001
Tumor size (cm)	3.60 (2.40–4.15)	3.1 (2.2–4.8)	3.4 (2.3–5.5)	4 (2.5–7.0)	5 (3.0–9.1)	< 0.001
Histology grade (I and II/III and IV)	688/303 (69.4%/30.6%)	284/97 (74.5%/25.5%)	61/30 (67.0%/33.0%)	241/119 (66.9%/33.1%)	102/57 (64.2%/35.8%)	0.045
Microscopic small vessel invasion (−/+)	498/493 (50.3%/49.7%)	209/172 (54.9%/45.1%)	49/42 (53.8%/46.2%)	173/187 (48.1%/51.9%)	67/92 (42.1%/57.9%)	0.036
Ishak modified HAI score (0–6/7–12)	571/420 (57.6%/42.4%)	216/165 (56.7%/43.3%)	38/53 (41.8%/58.2%)	226/134 (62.8%/37.2%)	91/68 (57.2%/42.8%)	0.004
Ishak fibrosis stage (0–4/5–6)	645/346 (65.1%/34.9%)	242/139 (63.5%/36.5%)	40/51 (44.0%/56.0%)	269/91 (74.7%/25.3%)	94/65 (59.1%/40.9%)	< 0.001
Steatosis (+/−)	499/492 (50.4%/49.6%)	381/0 (100%/0%)	91/0 (100%/0%)	16/344 (4.4%/95.6%)	11/148 (6.9%/93.1%)	< 0.001
Steatohepatitis (+)	110/881 (11.1%/88.9%)	89/292 (23.4%/76.6%)	17/74 (18.7%/81.3%)	1/359 (0.3%/99.7%)	3/156 (1.9%/98.1%)	< 0.001
Etiology						
HBsAg (+/−)	521/470 (52.6%/47.4%)	201/180 (47.2%/52.8%)	40/51 (44.0%/56.0%)	201/159 (55.8%/44.2%)	79/80 (49.7%/50.3%)	0.189
Anti‐HCV (+/−)	190/801 (19.2%/80.8%)	56/325 (14.7%/85.3%)	24/67 (26.4%/73.6%)	75/285 (20.8%/79.2%)	35/124 (22.0%/78.0%)	0.024
Serum biomarkers						
Platelet (mm^3^)	174,000 (133000–223,500)	176,000 (138000–218,000)	135,000 (88500–208,000)	182,000 (145700–227,000)	157,000 (103500–227,000)	< 0.001
INR	1.05 (1.01–1.11)	1.04 (1.00–1.09)	1.08 (1.02–1.15)	1.05 (1.01–1.10)	1.09 (1.04–1.15)	< 0.001
Albumin (g/dL)	4.1 (3.8–4.3)	4.2 (4.0–4.5)	3.6 (3.3–3.7)	4.2 (4.0–4.4)	3.6 (3.5–3.8)	< 0.001
Total bilirubin (mg/dL)	0.70 (0.52–0.97)	0.70 (0.52–0.96)	0.76 (0.54–1.04)	0.68 (0.51–0.90)	0.73 (0.52–1.10)	0.082
Creatinine (mg/dL)	0.84 (0.73–1.04)	0.86 (0.74–1.07)	0.80 (0.69–1.04)	0.84 (0.73–1.03)	0.82 (0.71–1.10)	0.629
ALT (U/l)	29.0 (19.0–47.0)	31.0 (22.0–40.0)	37.0 (23.0–55.5)	26.0 (18.0–41.0)	30.0 (19.0–57.5)	< 0.001
AST (U/l)	31.0 (23.0–46.0)	68.0 (40.0–152.0)	36.0 (27.0–56.5)	30.0 (22.0–45.0)	40.0 (28.0–69.5)	< 0.001
AFP (ng/mL)	10.8 (3.1–168.0)	7.3 (3.0–78.5)	10.5 (2.0–158.0)	16.8 (3.1–204.0)	23.4 (3.9–434.0)	0.001
Serum marker scores						
ALBI grade (1/2/3)	855/71/65 (86.3%/7.2%/6.6%)	338/27/16 (88.7%/7.1%/4.2%)	69/8/14 (75.8%/8.8%/15.4%)	328/22/10 (91.1%/6.1%/2.8%)	120/14/25 (75.5%/8.8%/15.7%)	< 0.001
FIB‐4	2.26 (1.51–3.54)	1.97 (1.39–2.85)	3.20 (2.00–6.22)	2.08 (1.47–3.02)	3.73 (2.52–5.34)	< 0.001
Cardiometabolic risk factors						
BMI > 23	672 (67.8%)	341 (89.5%)	71 (78.0%)	187 (51.9%)	73 (45.9%)	< 0.001
Hypertension	512 (51.7%)	218 (57.2%)	53 (58.2%)	158 (43.9%)	83 (52.2%)	< 0.001
Dyslipidaemia	401 (40.5%)	211 (55.4%)	40 (44.0%)	102 (28.3%)	48 (30.2%)	< 0.001
Diabetes	371 (37.4%)	188 (49.3%)	44 (48.4%)	96 (26.7%)	43 (27.0%)	< 0.001

*Note:* Group 1: MASLD, high PNI; Group 2: MASLD, low PNI; Group 3: no‐MASLD, high PNI; Group 4: no‐MASLD, low PNI.

Abbreviations: AFP, alpha‐fetoprotein; ALBI, albumin‐bilirubin; ALT, alanine transaminase; AST, aspartate transaminase; BMI, body mass index; FIB‐4, fibrosis‐4; INR, international normalized ratio.

### 3.2 Survival Status in Different Subgroups

**FIGURE 2:**
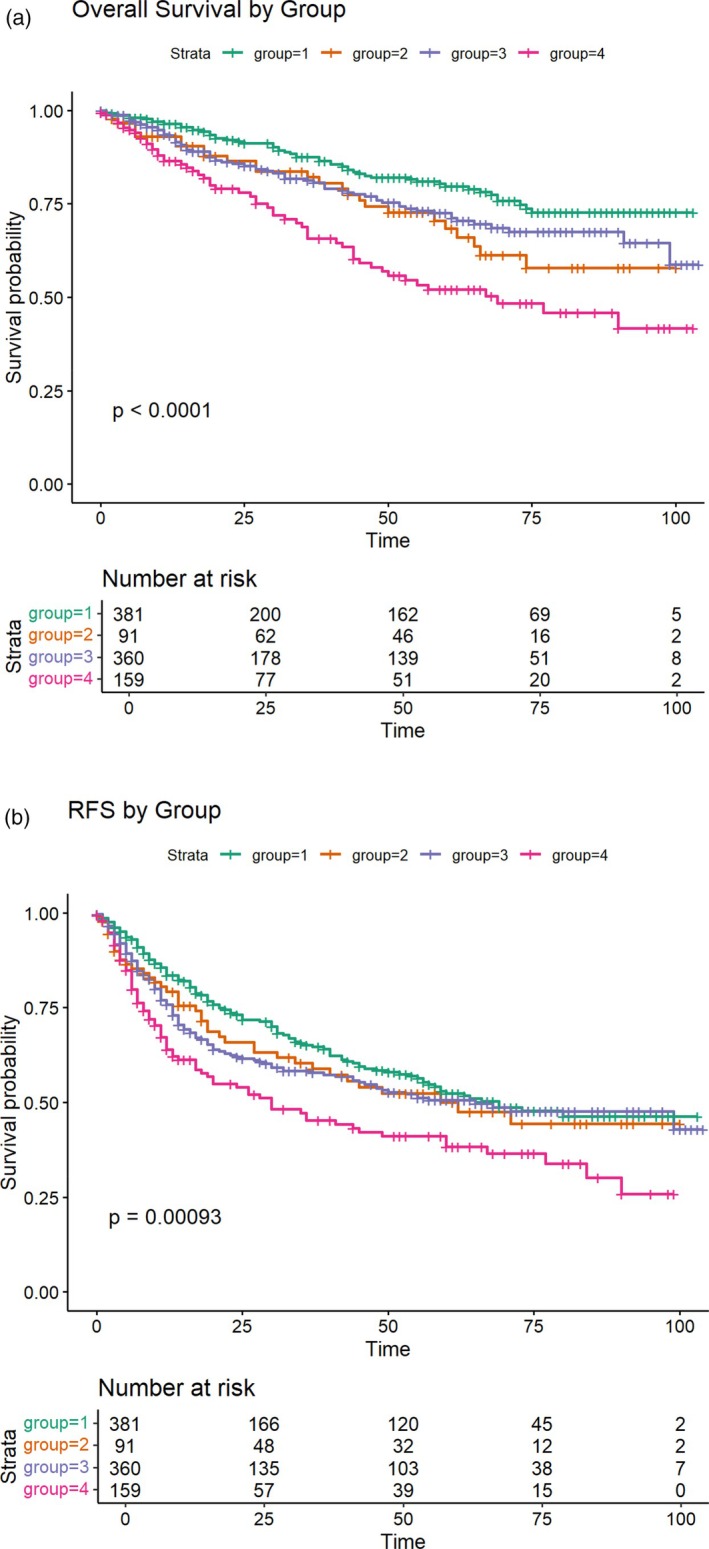
Kaplan–Meier survival analysis for (A) OS and (B) RFS of the four groups of patients stratified by MASLD and PNI status.

After a median follow‐up of 38.0 months (IQR 10.0–68.0 months), 207 patients died, and 287 patients had tumor recurrence after surgery. The overall 5‐year OS was 71.4%. In the four subgroups, the 5‐year OS rates were 79.7%, 68.3%, 72.5%, and 52.1% (Figure 2A). The overall 5‐year RFS was 49.3%, and the RFS rates of groups 1 through 4 were 52.4%, 50.0%, 50.6%, and 38.3%, respectively (Figure 2B). Patients without MASLD and low PNI (group 4) had significantly poorer OS and RFS than the other groups.

### 3.3 Risk Factors and Protective Factors

Univariate and multivariate analysis using the Cox proportional hazards model identified several factors associated with OS and RFS. Independent risk factors for OS included advanced tumor stage, microscopic vessel invasion, fibrosis‐4 (FIB‐4) grade, and serum levels of AST and AFP. A high PNI was identified as an independent protective factor against poor OS. MASLD showed significance in the univariate analysis but was not statistically significant after multivariate adjustment.

The independent risk factors identified for RFS were advanced tumor stage, microscopic vessel invasion, comorbid hypertension, FIB‐4 grade, and serum AST and AFP levels. The effect of MASLD and PNI on RFS was not statistically significant after multivariate adjustment. Detailed hazard ratios (HRs) and 95% confidence intervals (CIs) are shown in Table 2 (OS) and Table 3 (RFS). Since albumin was long identified as an important prognostic factor, another multivariate COX hazards model for OS and RFS including albumin instead of PNI was performed (Tables S1 and S2), demonstrating a better prognostic performance of PNI instead of using albumin alone.

**TABLE 2:** Univariate and multivariate COX proportional hazards models for OS of all enrolled patients.

Parameter	Univariate, HR (95% CI)	*p*	Multivariate, HR (95% CI), Model 1: PNI	*p*
Age > 65	1.48 (1.12–1.95)	0.005		
Sex (F)	0.98 (0.72–1.33)	0.881		
HBsAg (+)	0.85 (0.65–1.11)	0.236		
Anti‐HCV (+)	0.83 (0.57–1.19)	0.306		
Advanced stage	2.91 (2.20–3.83)	< 0.001	2.16 (1.58–2.97)	< 0.001
Multiple tumor	2.64 (2.01–3.47)	< 0.001		
Size > 5 cm	2.74 (2.08–3.60)	< 0.001		
Microscopic vessel invasion (+)	2.23 (1.66–3.00)	< 0.001	1.40 (1.00–1.96)	0.048
High histology grade	1.62 (1.23–2.13)	< 0.001		
ISHAK grade > 4	0.97 (0.72–1.29)	0.821		
HAI score > 6	1.03 (0.78–1.35)	0.850		
Platelet < 100,000	0.91 (0.68–1.21)	0.503		
INR > 1.1	1.34 (1.00–1.79)	0.047		
ALT > 40 (U/L)	1.25 (0.94–1.65)	0.123		
AST > 45 (U/L)	2.10 (1.59–2.77)	< 0.001	1.36 (1.01–1.83)	0.046
BILI > 1 (mg/dL)	1.06 (0.78–1.44)	0.721		
Creatinine > 1.2 (mg/dL)	1.68 (1.19–2.35)	0.003		
AFP > 20 (ng/mL)	1.89 (1.43–2.49)	< 0.001	1.38 (1.02–1.87)	0.034
ALBI grade 2 or 3	1.22 (0.85–1.75)	0.288		
FIB4 grade 2 or 3	1.63 (1.13–2.36)	0.009	1.54 (1.04–2.26)	0.030
PNI > 45	0.52 (0.40–0.69)	< 0.001	0.65 (0.49–0.87)	0.004
BMI > 23	0.73 (0.55–0.97)	0.027		
Type 2 DM	0.98 (0.73–1.30)	0.876		
HTN	1.36 (1.03–1.78)	0.030		
Dyslipidaemia	0.98 (0.74–1.30)	0.876		
MASLD (+)	0.62 (0.47–0.82)	< 0.001		
MASH (+)	1.17 (0.77–1.80)	0.459		

Abbreviations: AFP, alpha‐fetoprotein; ALBI, albumin‐bilirubin; ALT, alanine transaminase; AST, aspartate transaminase; BMI, body mass index; FIB‐4, fibrosis‐4; HTN, hypertension; INR, international normalized ratio; MASH, metabolic dysfunction‐associated steatohepatitis; MASLD, metabolic dysfunction‐associated steatotic liver disease; PNI, Prognostic Nutritional Index.

**TABLE 3:** Univariate and multivariate COX proportional hazards models for RFS of all enrolled patients.

Parameter	Univariate, HR (95% CI)	*p*	Multivariate, HR (95% CI), Model 1: PNI	*p*
Age > 65	1.19 (0.98–1.46)	0.085		
Sex (F)	1.09 (0.87–1.36)	0.442		
HBsAg (+)	1.00 (0.82–1.22)	0.994		
Anti‐HCV (+)	1.09 (0.85–1.40)	0.501		
Advanced stage	2.36 (1.93–2.89)	< 0.001	1.87 (1.49–2.35)	< 0.001
Multiple tumor	1.93 (1.58–2.37)	< 0.001		
Size > 5 cm	2.06 (1.69–2.52)	< 0.001		
Microscopic vessel invasion (+)	2.05 (1.66–2.53)	< 0.001	1.47 (1.16–1.87)	0.001
High histology grade	1.29 (1.05–1.59)	0.014		
ISHAK grade > 4	1.07 (0.87–1.32)	0.540		
HAI score > 6	1.11 (0.91–1.36)	0.312		
Platelet < 100,000	0.96 (0.78–1.19)	0.725		
INR > 1.1	1.28 (1.03–1.59)	0.025	1.25 (1.00–1.55)	0.048
ALT > 40 (U/L)	1.36 (1.11–1.67)	0.004		
AST > 45 (U/L)	1.76 (1.42–2.17)	< 0.001	1.30 (1.04–1.63)	0.023
BILI > 1 (mg/dL)	0.96 (0.76–1.21)	0.725		
Creatinine > 1.2 (mg/dL)	1.14 (0.86–1.49)	0.360		
AFP > 20 (ng/mL)	1.49 (1.22–1.82)	< 0.001	1.28 (1.04–1.57)	0.019
ALBI grade 2 or 3	1.14 (0.86–1.50)	0.357		
FIB4 grade 2 or 3	1.32 (1.02–1.71)	0.034	1.31 (1.01–1.72)	0.046
PNI > 45	0.72 (0.58–0.89)	0.003		
BMI > 23	0.89 (0.72–1.09)	0.264		
Type 2 DM	1.01 (0.82–1.24)	0.933		
HTN	1.32 (1.08–1.61)	0.007	1.30 (1.06–1.59)	0.012
Dyslipidaemia	0.97 (0.79–1.20)	0.803		
MASLD (+)	0.76 (0.62–0.93)	0.008		
MASH (+)	1.05 (0.76–1.46)	0.755		

Abbreviations: AFP, alpha‐fetoprotein; ALBI, albumin‐bilirubin; ALT, alanine transaminase; AST, aspartate transaminase; BMI, body mass index; FIB‐4, fibrosis‐4; HTN, hypertension; INR, international normalized ratio; MASH, metabolic dysfunction‐associated steatohepatitis; MASLD, metabolic dysfunction‐associated steatotic liver disease; PNI, Prognostic Nutritional Index.

### 3.4 Subgroups With and Without Concurrent MASLD

**FIGURE 3:**
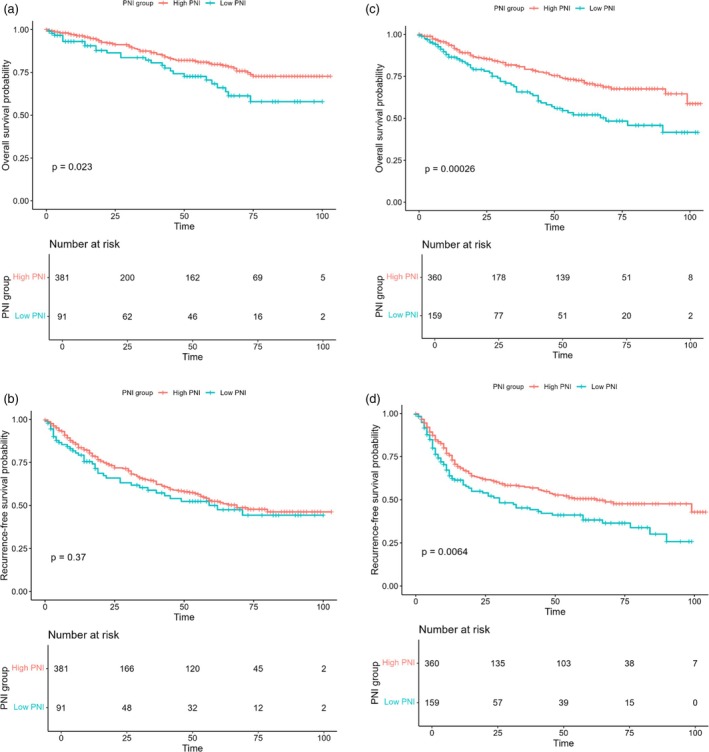
Kaplan–Meier survival analysis stratified by PNI for (A) OS, (B) RFS in the MASLD subgroup and (C) OS, (D) RFS in the no‐MASLD subgroup.

We divided the patients into two groups according to the presence of MASLD. In the cohort of patients with HCC and concurrent MASLD, KM survival analysis indicated better OS among patients with higher PNI, while no significant difference was noted for RFS (Figure 3A,B). Univariate and multivariate COX analysis indicated that advanced tumor stage, high FIB‐4 grade, presence of MASH, and high histology grade were independent risk factors for poor OS, while advanced tumor stage, high FIB‐4 grade, and high serum AFP were independent risk factors for poor RFS.

Conversely, in the cohort of patients with HCC without concurrent MASLD, KM survival analysis indicated better OS and RFS among patients with higher PNI (Figure 3C,D). Univariate and multivariate COX analysis indicated that high PNI was a protective factor for poor OS, and multifocal disease was an independent risk factor. Advanced tumor stage, high serum AST levels, microscopic vessel invasions, and comorbid hypertension were independent risk factors for poor RFS (Table 4).

**TABLE 4:** Multivariate COX proportional hazards models for OS/RFS in the subgroup of patients with or without MASLD.

Parameter	HR (95% CI)	*p*
OS: MASLD		
Advanced stage	3.27 (2.07–5.17)	< 0.001
FIB‐4 grade 2 or 3	2.35 (1.25–4.40)	0.008
MASH (+)	2.23 (1.35–3.70)	0.002
High histology grade	1.79 (1.09–2.93)	0.022
OS: no‐MASLD		
PNI > 45	0.59 (0.41–0.84)	0.003
Multiple tumors	1.71 (1.02–2.89)	0.044
RFS: MASLD		
Advanced stage	2.21 (1.62–3.00)	< 0.001
FIB‐4 grade 2 or 3	1.66 (1.13–2.45)	0.010
AFP > 20 (ng/mL)	1.51 (1.11–2.04)	0.009
RFS: no‐MASLD		
Advanced stage	1.81 (1.34–2.45)	< 0.001
Microscopic vessel invasion (+)	1.71 (1.24–2.35)	0.001
Hypertension	1.51 (1.15–1.98)	0.003
AST > 45 (U/L)	1.36 (1.03–1.81)	0.033

Abbreviations: AFP, alpha‐fetoprotein; AST, aspartate transaminase; FIB‐4, fibrosis‐4; MASH, metabolic dysfunction‐associated steatohepatitis.

### 3.5 Sensitivity Analysis

Due to a significantly higher PNI in the MASLD subgroup (mean PNI 50.2 vs. 47.9, *p <* 0.001), we re‐stratified the patients in the MASLD subgroup according to a PNI cut‐off of 50, which was approximate to the median PNI in this subgroup. This was done to address potential analytical bias. KM analysis and multivariate Cox regression were performed again. The KM analysis using the new PNI cut‐off showed borderline significance in regard to OS, but PNI was still not identified as a significant risk factor for either OS or RFS (Table S3 and Figure S1).

### 3.6 Subgroup Analysis by Viral Etiology

Due to a substantial amount of our patients having chronic viral hepatitis B or C (CHB, *n* = 521 or CHC, *n* = 190), subgroup KM survival analysis stratified by viral hepatitis status for OS and RFS was performed (Figures S2 and S3). For patients with CHB, a better nutritional status was associated with better OS and RFS in the non‐MASLD group but not in the MASLD group, which was consistent with the general cohort. In HCC patients without viral etiologies (*n* = 306), a trend of better OS in the well‐nutritional cohort was observed regardless of the presence of MASLD, while no statistically significant differences in RFS were found. For CHC patients, the role of nutritional status and MASLD on the outcome of HCC was limited.

### 3.7 The Role of PNI on Early and Late Recurrence

To further investigate the role of PNI on early (recurrence in 2 years after surgery) and late recurrence, KM survival analysis was conducted (Figures S4 and S5). For early recurrence, PNI has no stratifying ability no matter for patients with or without MASLD. However, PNI showed stratifying ability for late recurrence in patients both with and without MASLD.

## 4 Discussion

There were several notable findings in this study. First, stratification of HCC patients according to concurrent MASLD and nutritional status demonstrated a significant difference in outcomes between groups. The presence of MASLD and high PNI were associated with the most favorable outcomes for patients with HCC undergoing surgical resection. Second, high PNI was identified as an independent protective factor in the overall HCC cohort and in patients with HCC without concurrent MASLD, but not in patients with both HCC and concurrent MASLD. Moreover, the risk factors identified for OS and RFS were different across the general cohort and subgroups. More tumor factors were identified for RFS, while more additional field factors were identified for OS [21, 22, 23, 24]. Interestingly, while evaluating recurrence by early and late, the role of nutritional status appeared to be more significant in late recurrence. The finding was well correlated with previous studies, while late recurrence was more correlated with field factors compared to early recurrence, which was predominantly associated with tumor factors [25].

PNI was identified as a significant prognostic factor for all patients with HCC, which is consistent with previous studies [12, 13]. In subgroup analyses, PNI was a significant protective factor in the no‐MASLD group but not in the MASLD group. Interestingly, a significantly higher PNI value was noted in the MASLD cohort compared to the no‐MASLD cohort, which may potentially indicate better nutritional status in patients with concurrent MASLD. However, despite applying an adjusted higher cut‐off, PNI was still not identified as a significant factor for either OS or RFS in the MASLD subgroup. This lack of association may have been caused by generally better nutritional status in patients with concurrent MASLD and suggests that tumor factors may play more important roles in the prognosis for this specific subgroup of patients.

Nutritional status plays an important role in the prognosis of patients with cancer and especially HCC [12]. The manifestation of malnutrition can vary and can include reduced hand grip strength and decreased muscle mass. Van Dijk et al. demonstrated that reduced hand grip strength is an indicator for poor outcome among patients with HCC [26]. Lee et al. also demonstrated that changes in muscle mass and body composition are important factors for the outcome of patients with HCC following surgical resection [27]. Several molecular mechanisms have also been proposed, including impaired lipoprotein synthesis and an altered gut–liver axis [28]. However, due to the lack of a single direct measurement or indicator for nutritional status, standardized nutritional care has been difficult to achieve.

Inconsistencies across different nutritional studies on patients with cancer were noted, which make it difficult to translate research findings into clinical practice [29]. To address this need, PNI has been widely accepted as an indicator for nutritional status and has been used to predict outcomes of various malignant and non‐malignant diseases [12, 30]. To the best of our knowledge, however, our study is the first to incorporate non‐invasive assessment of nutritional status with MASLD and HCC. PNI can be useful for clinicians because the score is calculated from a single blood test, which allows for easy assessment of nutritional status and good performance of risk stratifications. Moreover, sequential measurements are easily available, which can facilitate personalized clinical adjustments.

The present cohort showed significant differences in baseline characteristics across the four groups, including tumor characteristics, serum biochemistry, and fibrosis and inflammation status. This finding demonstrates the multisystemic impact of steatotic liver disease, CMRFs, and nutritional status. van Zutphen et al. found that malnutrition leads to severe impairments in hepatic peroxisomal and mitochondrial function, as well as hepatic metabolic dysfunction, which can lead to liver fibrosis or carcinogenesis [31].

Buyken et al. and Barrea et al. discussed the potential effects of nutrition on the liver–spleen axis, which are likely due to pro‐inflammatory cytokines, including interleukin (IL)‐1β and IL‐6, tumor necrosis factor (TNF)‐α, monocyte chemotactic protein‐1 (MCP‐1), and plasminogen activator inhibitor‐1 (PAI‐1) [32, 33]. These factors can cause systemic inflammation and have global impacts. As a result, metabolic dysfunction and nutrition do not simply contribute to steatosis but instead have systemic effects through insulin resistance, inflammation, and other pathways that affect patient outcomes.

The role of CMRF and SLD on the outcomes of patients with HCC has long been discussed with inconsistent results. In the present cohort, a higher proportion of obese patients was noted among the patients with concurrent MASLD. Obesity has been reported as a protective factor against poor outcomes, likely because of the reduced incidence of sarcopenia, which has long been identified as a risk factor for poor outcomes in patients with HCC [27, 34].

Interestingly, Shinkawa et al. investigated the combined effects of obesity and DM on the outcomes of patients with HCC and found that the co‐occurrence of obesity and DM was a risk factor for poor OS and late recurrence, while the sole presence of DM or obesity was not [35]. This finding demonstrates the diverse impacts of CMRFs on the outcomes of patients with HCC. In the present study, no single CMRF was identified as an independent risk factor for poor OS. This suggests that despite the well‐acknowledged importance of CMRFs, considerations of nutritional status and liver steatosis are equally important in assessing the risks and outcomes of patients.

Liver fibrosis and cirrhosis play important roles in the progression of liver diseases and the carcinogenesis of HCC. Interestingly, in the present study, the ISHAK fibrosis grade alone was not identified as an independent risk factor for poor OS or RFS. In contrast, serum‐based non‐invasive tests like FIB‐4 showed significance even after multivariable adjustments. This finding suggests that instead of solely reflecting fibrosis status, FIB‐4 has additional prognostic potential and may reflect the general liver condition of patients. A review by Lee et al. demonstrated the ability of FIB‐4 to predict risk and stratify patients for liver‐related morbidity and mortality [36]. Current guidelines also suggest incorporating non‐invasive tests into risk‐stratifying strategies to aid clinicians by providing a more precise, comprehensive tool to assess patient conditions [37].

MASLD can simultaneously be involved in the etiology and act as a field factor for liver diseases. Lim et al. observed a higher incidence of HCC among individuals with MASLD and advanced fibrosis [7]. Pinyol et al. found that C>T and C>A transitions were more frequent in cases of higher Wnt/transforming growth factor‐β (TGF‐β) molecular presentation, which was unique to the carcinogenesis of HCC with other etiologies [38].

On the other hand, while present as a field factor, the relationships between MASLD and other etiologies are multidirectional and diverse. For example, Huang et al. observed that MASLD was associated with decreased all‐cause mortality and liver‐related events in patients with chronic hepatitis B [9]. Han et al. also proposed possible mechanisms for elevated intrahepatic interferon‐related gene signatures in patients with chronic hepatitis B [39]. In the present cohort, we observed better OS and RFS in subgroups with MASLD, but MASLD itself was not identified as an independent protective factor after multivariable adjustment. In our subgroup analysis, PNI stratified the OS better in patients with single etiologies, especially CHB (Figure S2A) and “pure” MASLD (Figure S2F). On the other hand, its prognostic ability blunted in mixed etiologies. The interesting finding suggests the heterogenous disease driver in multi‐etiological cases. Therefore, MASLD and other etiologies, such as viral hepatitis and alcoholic liver disease, have diverse impacts. Both clinical relationships and molecular mechanisms require further investigation.

Despite the novel findings, there are several limitations to our study. The research was a single‐center retrospective cohort study, and the subgroup analysis involved limited patient numbers in each subgroup, which may diminish the association in specific subgroups. Despite the good performance of PNI for risk stratification and prognostication, it still lacks consideration of other important factors of nutritional status, such as body composition and muscle strength, and further causal relationship study regarding nutrition, body composition, and the outcome of HCC patients is still warranted. Also, most of our patients had underlying viral etiologies, and the ethnicities were predominantly East‐Asian. The effect of nutrition on other races or pure MASLD‐related HCC cannot be comprehensively assessed in our study. Furthermore, socio‐economic status, disease awareness, and ethnicity were not easily addressed or balanced.

In conclusion, as an indicator for nutritional and inflammatory status, the PNI serves as an important factor in differentiating patients with HCC undergoing surgical resection into different risk groups. High PNI is an independent protective factor against poor OS, especially in patients without concurrent MASLD.

## Funding

This work was supported by the National Science and Technology Council of Taiwan (NSTC 112‐2314‐B‐075‐043‐MY2, NSTC 114‐2314‐B‐075‐070, NSTC 114‐2622‐E‐075‐001), Taipei Veterans General Hospital (V113C‐130, V114C‐099), Centre of Excellence for Cancer Research (MOHW114‐TDU‐B‐221‐144007 and Big Data Centre), Y.L. Lin Hung Tai Education Foundation. The funders had no role in the design of the study; in the collection, analyses, or interpretation of data; in the writing of the manuscript; or in the decision to publish the results.

## Conflicts of Interest

The authors declare no conflicts of interest.

## Supporting information


**Figure S1:** Kaplan–Meier survival analysis for (A) OS, (B) RFS of the four groups of patients stratified by MASLD and PNI status using a higher cut‐off at 50.
**Figure S2:** Kaplan–Meier survival analysis for OS for stratified by PNI status of HCC patients with (A) CHB but without MASLD, (B) CHB and MASLD, (C) CHC without MASLD, (D) CHC and MASLD, (E) without MASLD and viral hepatitis (F) with MASLD but without viral hepatitis.
**Figure S3:** Kaplan–Meier survival analysis for RFS for stratified by PNI status of HCC patients with (A) CHB but without MASLD, (B) CHB and MASLD, (C) CHC without MASLD, (D) CHC and MASLD (E) without MASLD and viral hepatitis (F) with MASLD but without viral hepatitis.
**Figure S4:** Kaplan–Meier survival analysis for early recurrence stratified by PNI status of HCC patients (A) with MASLD (B) without MASLD.
**Figure S5:** Kaplan–Meier survival analysis for late recurrence stratified by PNI status of HCC patients (A) with MASLD (B) without MASLD.
**Table S1:** Univariate and multivariate COX proportional hazards models for OS of all enrolled patients, including albumin instead of PNI.
**Table S2:** Univariate and multivariate COX proportional hazards models for RFS of all enrolled patients, including albumin instead of PNI.
**Table S3:** Multivariate COX proportional hazardous models for OS/RFS in the subgroup of patients with MASLD using higher cut‐off of 50.

## Data Availability

The data that support the findings of this study are available on request from the corresponding author. The data are not publicly available due to privacy or ethical restrictions.
