# Supplementary material for: Interactive Roles of Metabolic Dysfunction‐Associated Steatotic Liver Disease and Nutritional Status in the Prognosis of Patients With Hepatocellular Carcinoma After Surgical Resection
Source: Kaohsiung J Med Sci. 2026 Jul 3:e70256. Online ahead of print. doi: 10.1002/kjm2.70256 (PMC13399853; doi:10.1002/kjm2.70256)
Supplement: Supplementary file 1 — Figure S1: Kaplan–Meier survival analysis for (A) OS, (B) RFS of the four groups of patients stratified by MASLD and PNI status using a higher cut‐off at 50. Figure S2: Kaplan–Meier survival analysis for OS for stratified by PNI status of HCC patients with (A) CHB but without MASLD, (B) CHB and MASLD, (C) CHC without MASLD, (D) CHC and MASLD, (E) without MASLD and viral hepatitis (F) with MASLD but without viral hepatitis. Figure S3: Kaplan–Meier survival analysis for RFS for stratified by PNI status of HCC patients with (A) CHB but without MASLD, (B) CHB and MASLD, (C) CHC without MASLD, (D) CHC and MASLD (E) without MASLD and viral hepatitis (F) with MASLD but without viral hepatitis. Figure S4: Kaplan–Meier survival analysis for early recurrence stratified by PNI status of HCC patients (A) with MASLD (B) without MASLD. Figure S5: Kaplan–Meier survival analysis for late recurrence stratified by PNI status of HCC patients (A) with MASLD (B) without MASLD. Table S1: Univariate and multivariate COX proportional hazards models for OS of all enrolled patients, including albumin instead of PNI. Table S2: Univariate and multivariate COX proportional hazards models for RFS of all enrolled patients, including albumin instead of PNI. Table S3: Multivariate COX proportional hazardous models for OS/RFS in the subgroup of patients with MASLD using higher cut‐off of 50. [file KJM2-9999-e70256-s001.docx]

**Supplementary legends**

**SUPPLEMENTARY FIGURE S1 |** Kaplan–Meier survival analysis for (A) OS (B) RFS of the four groups of patients stratified by MASLD and PNI status using a higher cut-off at 50.

**SUPPLEMENTARY FIGURE S2 |** Kaplan–Meier survival analysis for OS for stratified by PNI status of HCC patients with (A) CHB but without MASLD (B) CHB and MASLD (C) CHC without MASLD (D) CHC and MASLD (E)without MASLD and viral hepatitis (F) with MASLD but without viral hepatitis

**SUPPLEMENTARY FIGURE S3 |** Kaplan–Meier survival analysis for RFS for stratified by PNI status of HCC patients with (A) CHB but without MASLD (B) CHB and MASLD (C) CHC without MASLD (D) CHC and MASLD (E)without MASLD and viral hepatitis (F) with MASLD but without viral hepatitis

**SUPPLEMENTARY FIGURE S4 |** Kaplan–Meier survival analysis for early recurrence stratified by PNI status of HCC patients (A) with MASLD (B) without MASLD

**SUPPLEMENTARY FIGURE S5 |** Kaplan–Meier survival analysis for late recurrence stratified by PNI status of HCC patients (A) with MASLD (B) without MASLD

**SUPPLEMENTARY TABLE S1 |** Univariate and multivariate COX proportional hazards models for OS of all enrolled patients, including albumin instead of PNI.

**SUPPLEMENTARY TABLE S2 |** Univariate and multivariate COX proportional hazards models for RFS of all enrolled patients, including albumin instead of PNI.

**SUPPLEMENTARY TABLE S3 |** Multivariate COX proportional hazardous models for OS/RFS in the subgroup of patients with MASLD using higher cut-off of 50.

**SUPPLEMENTARY FIGURE S1(A).**

**
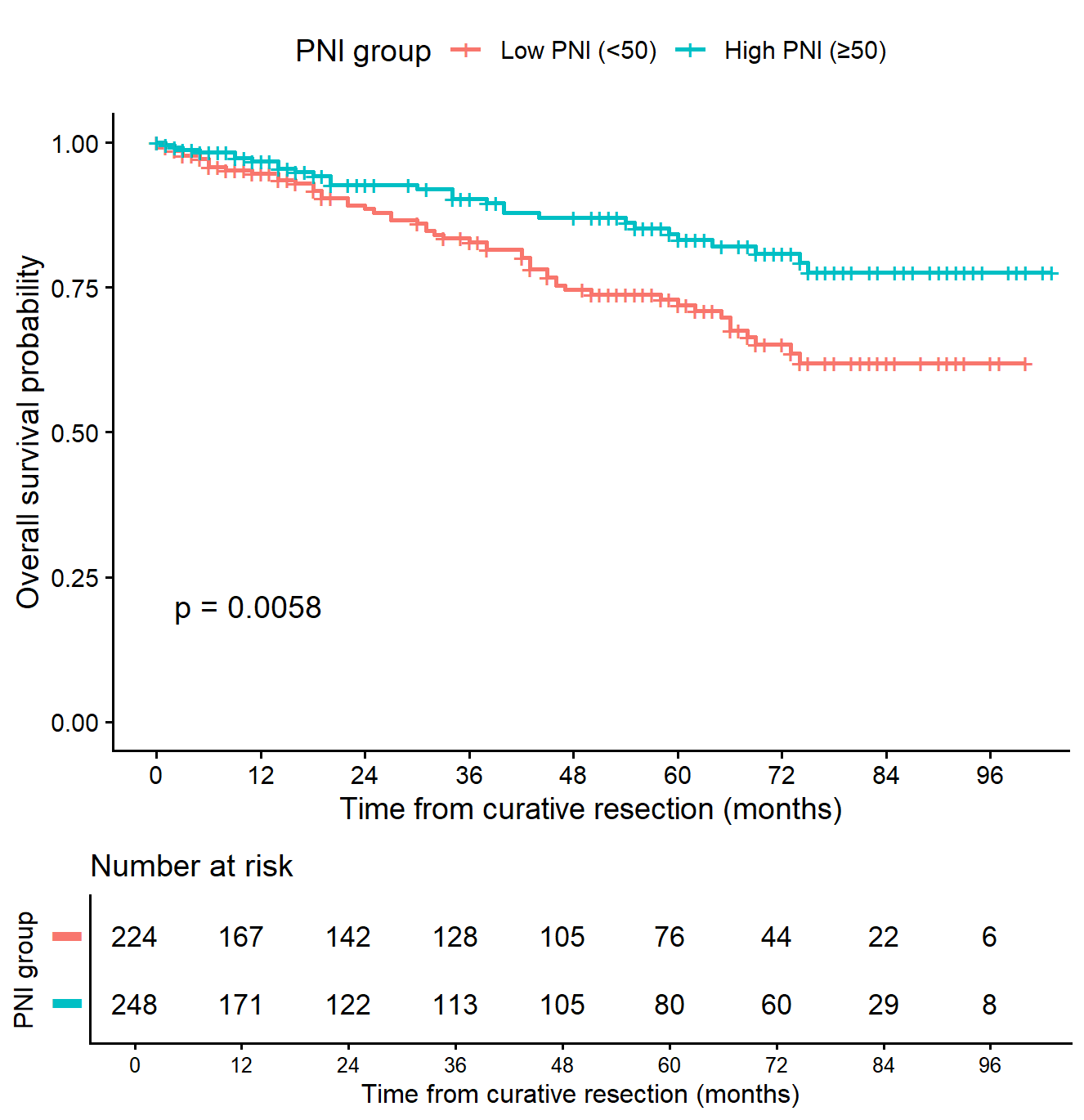
**

**SUPPLEMENTARY FIGURE S1(B).**


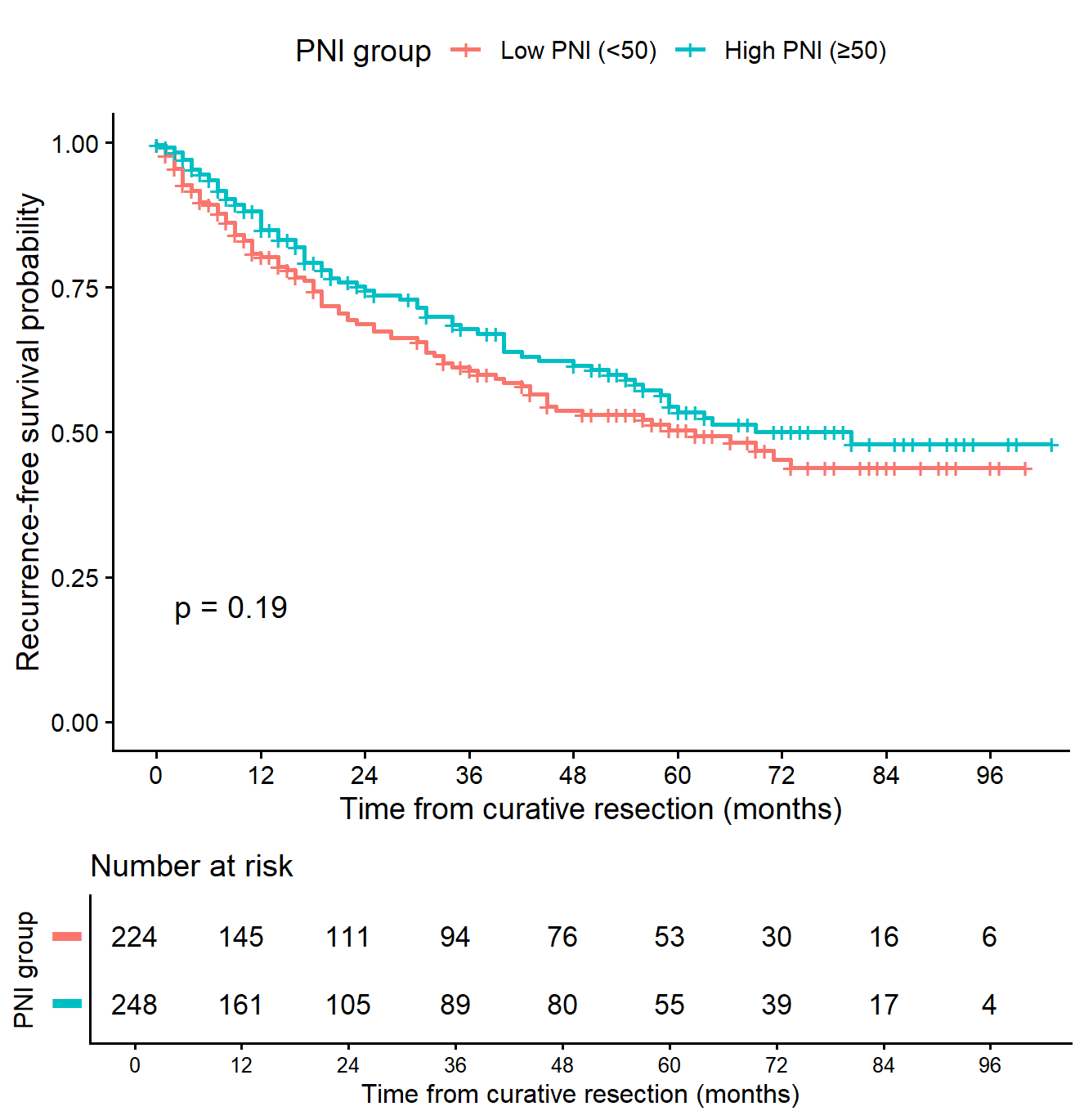


**SUPPLEMENTARY FIGURE S2(A).**


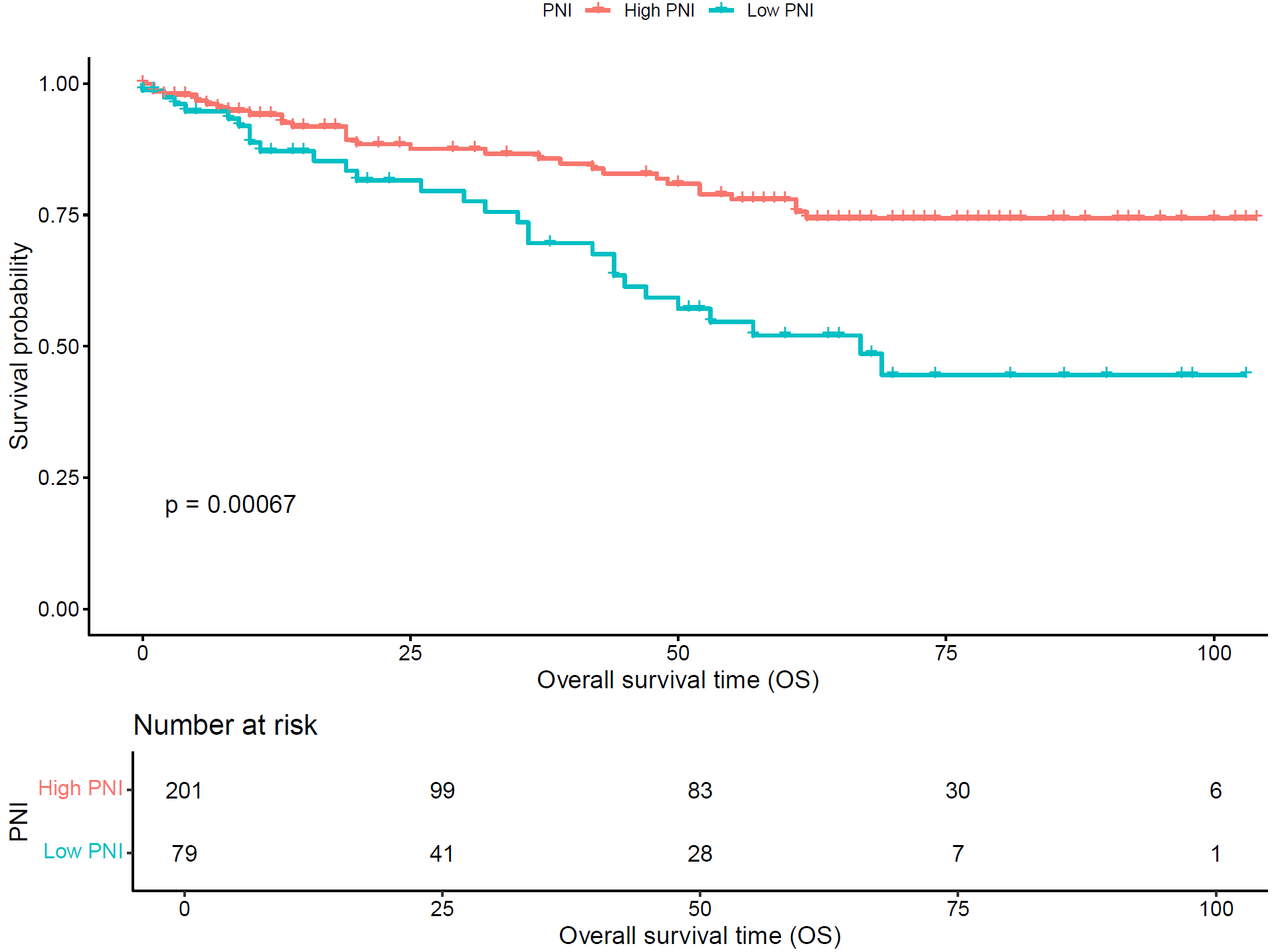


**SUPPLEMENTARY FIGURE S2(B).**


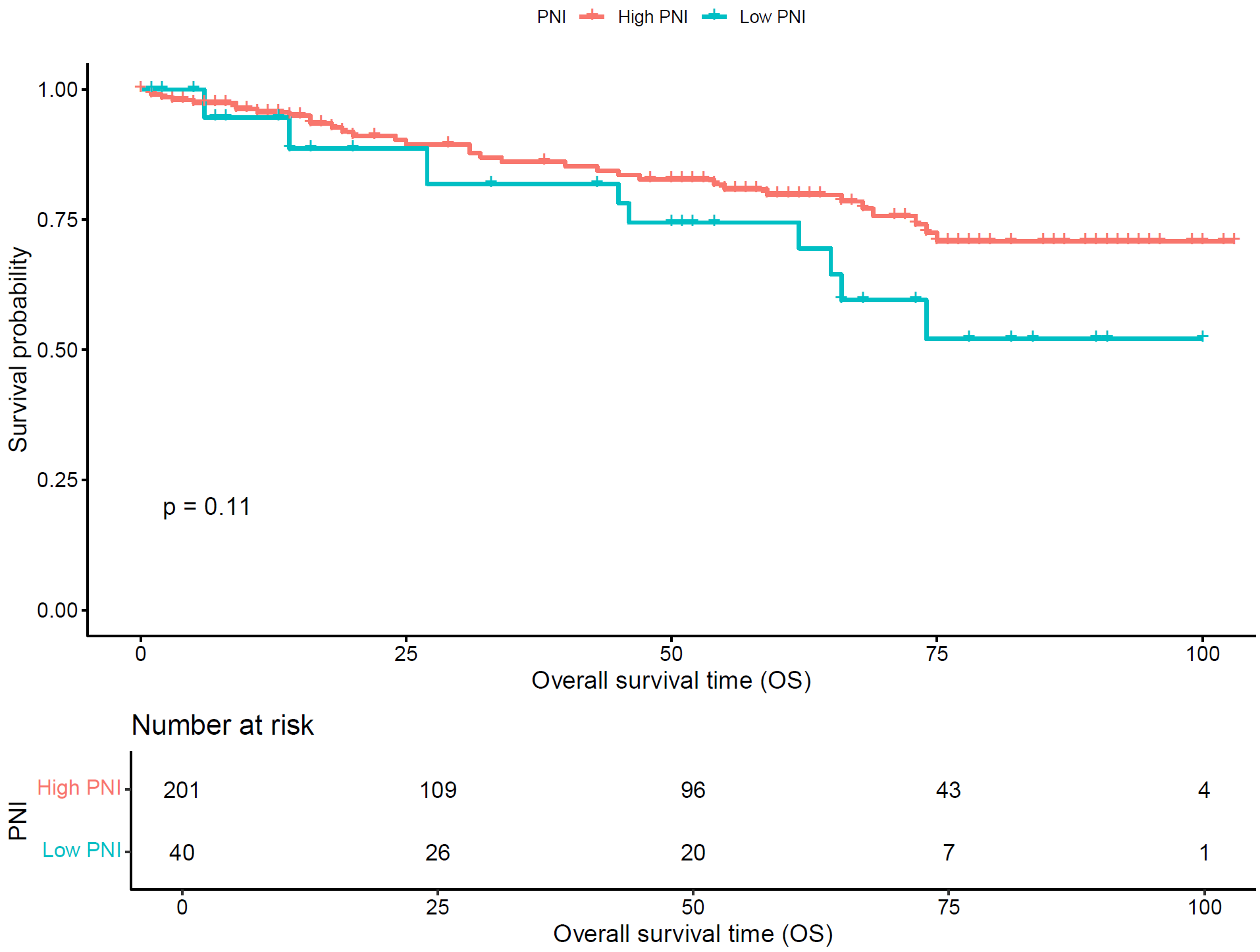


**SUPPLEMENTARY FIGURE S2(C).**


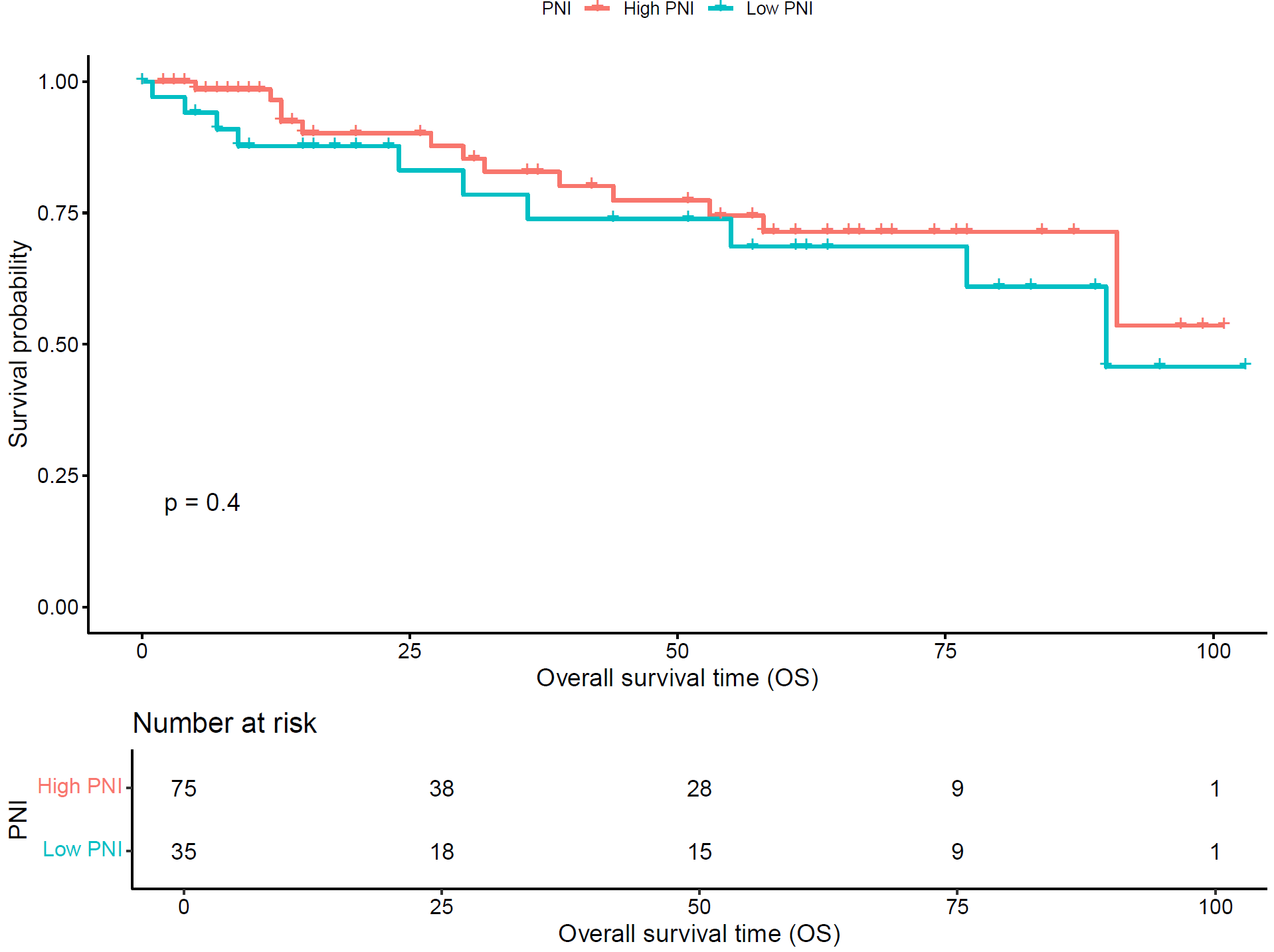


**SUPPLEMENTARY FIGURE S2(D).**


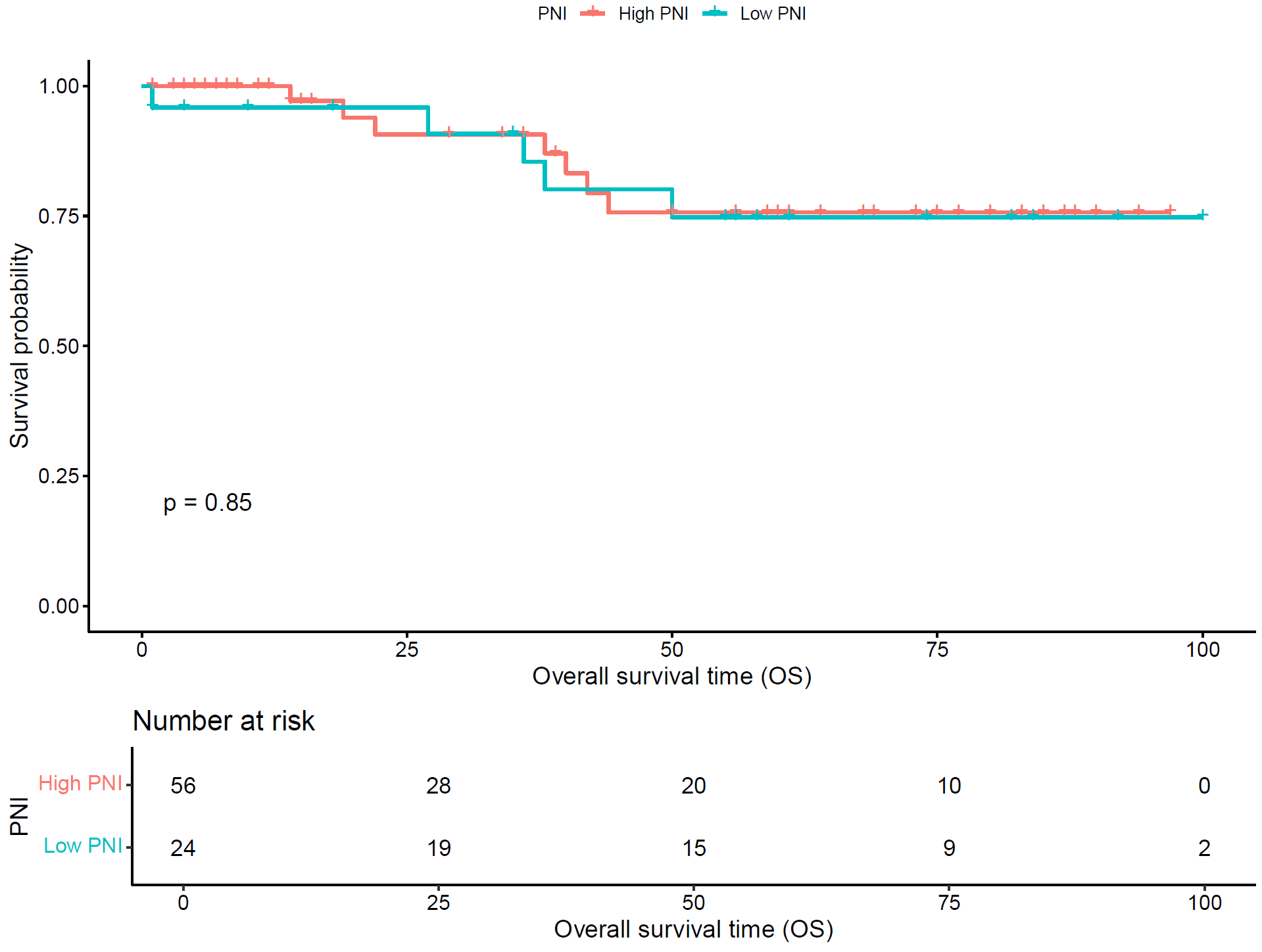


**SUPPLEMENTARY FIGURE S2(E).**

**
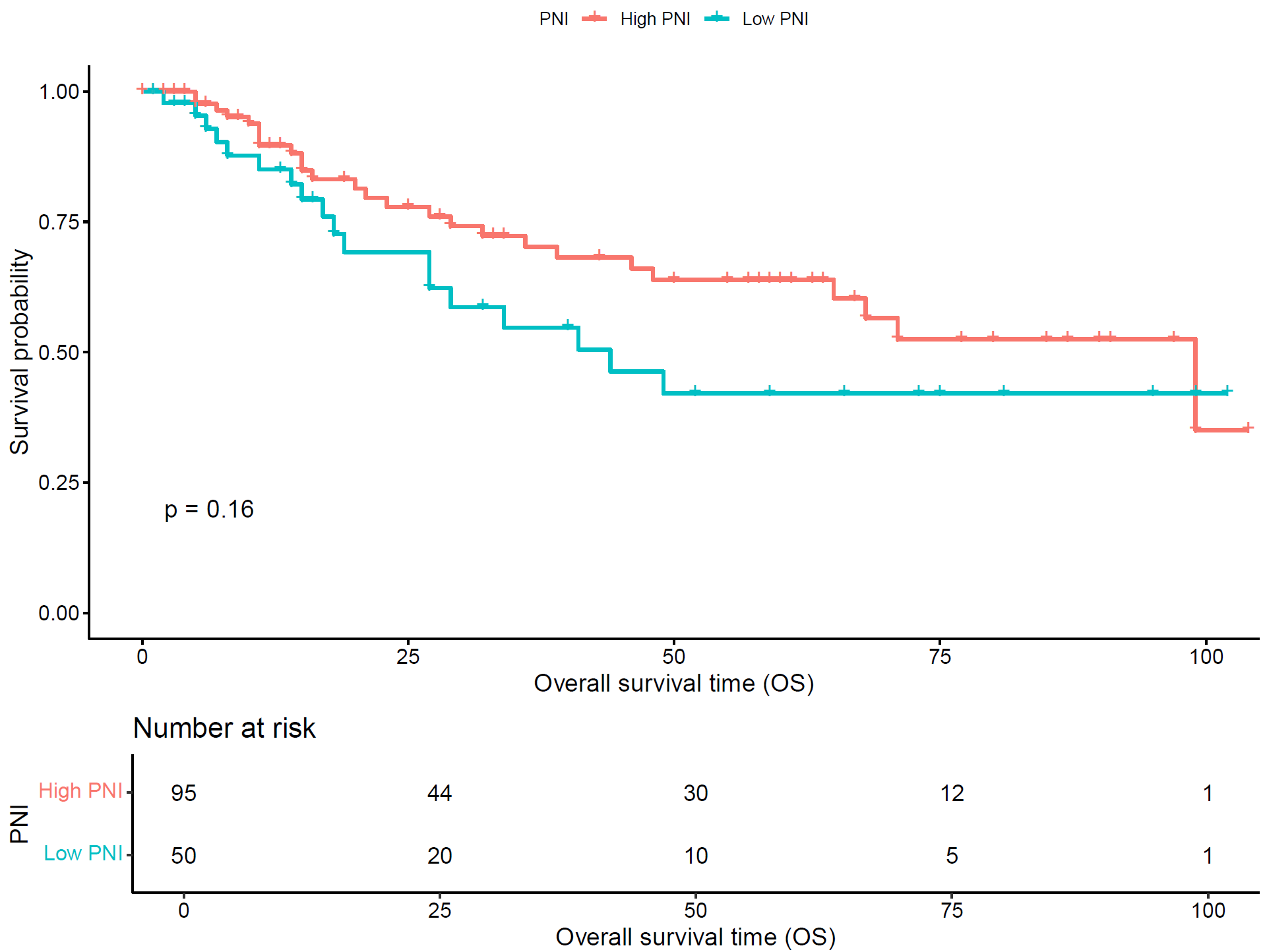
**

**SUPPLEMENTARY FIGURE S2(F).**

**
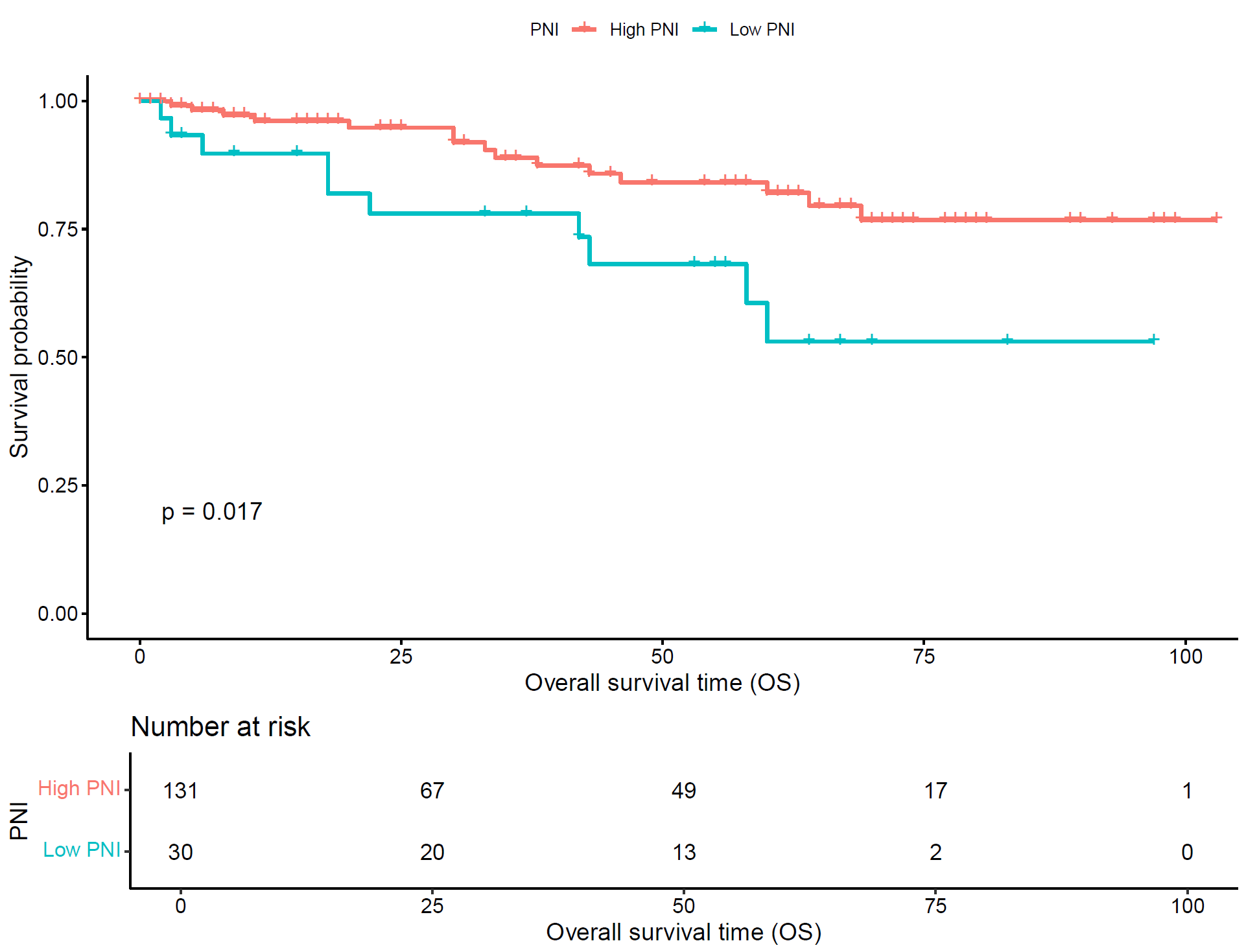
**

**SUPPLEMENTARY FIGURE S3(A).**

**
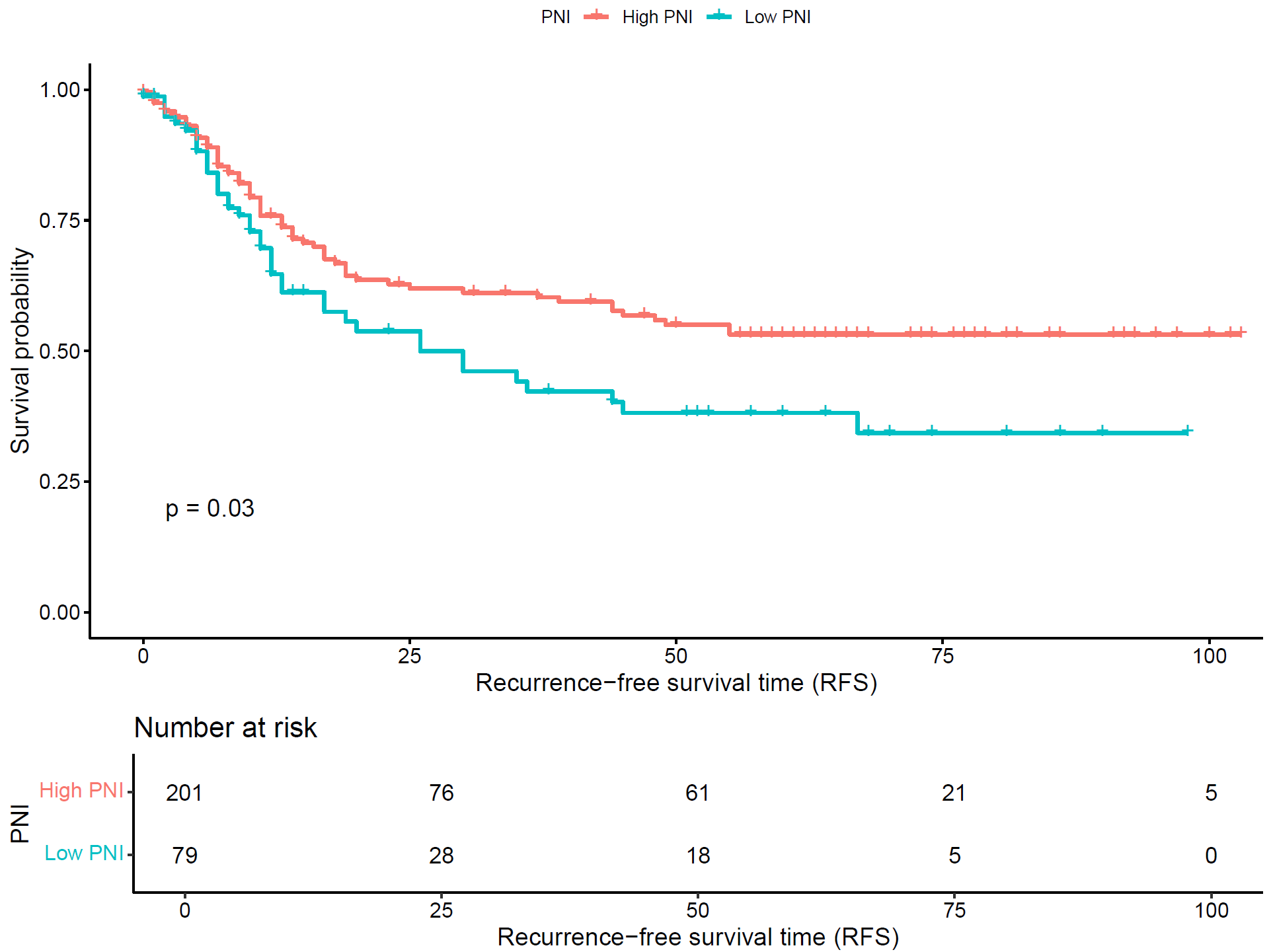
**

**SUPPLEMENTARY FIGURE S3(B).**

**
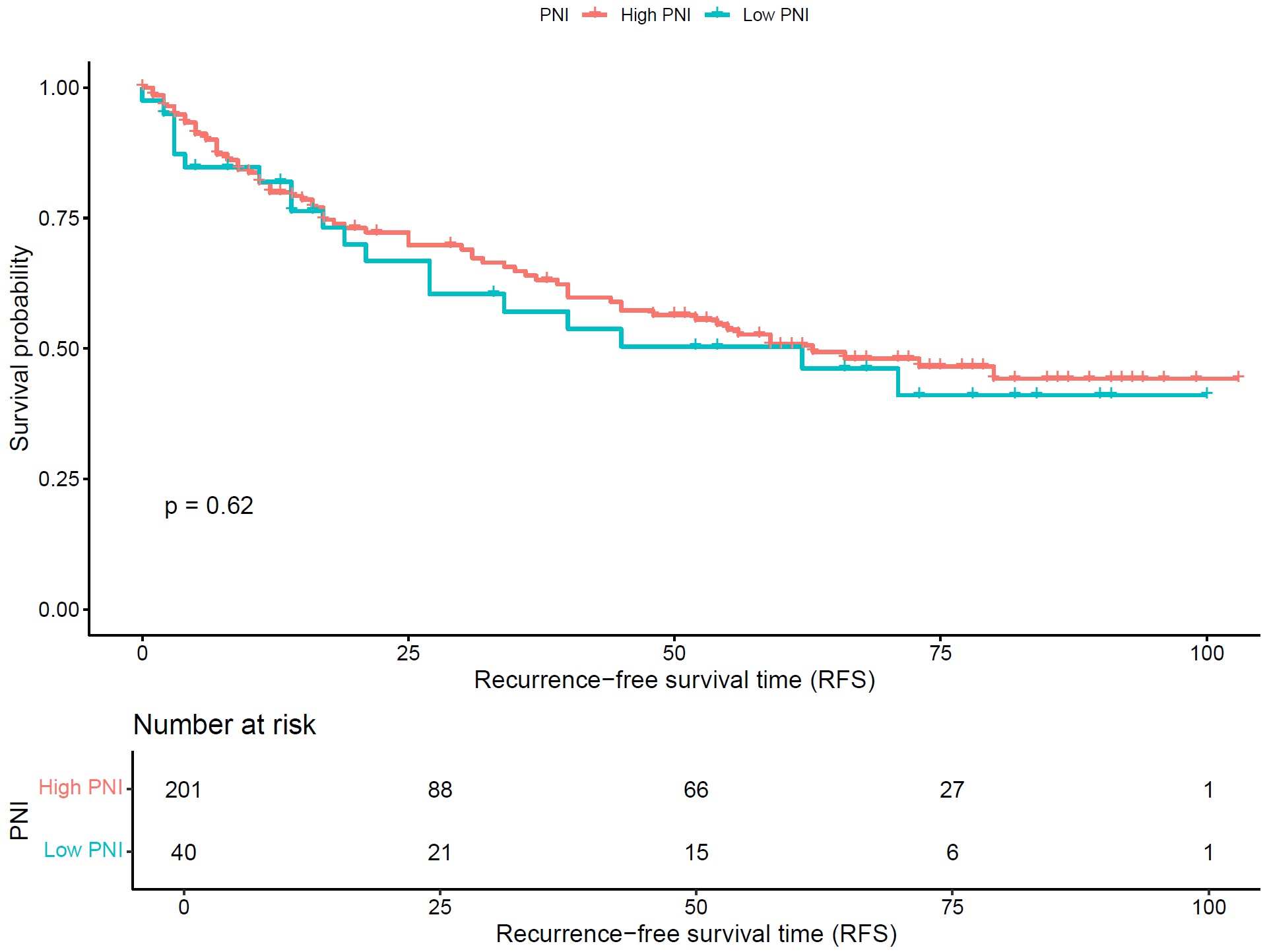
**

**SUPPLEMENTARY FIGURE S3(C).**

**
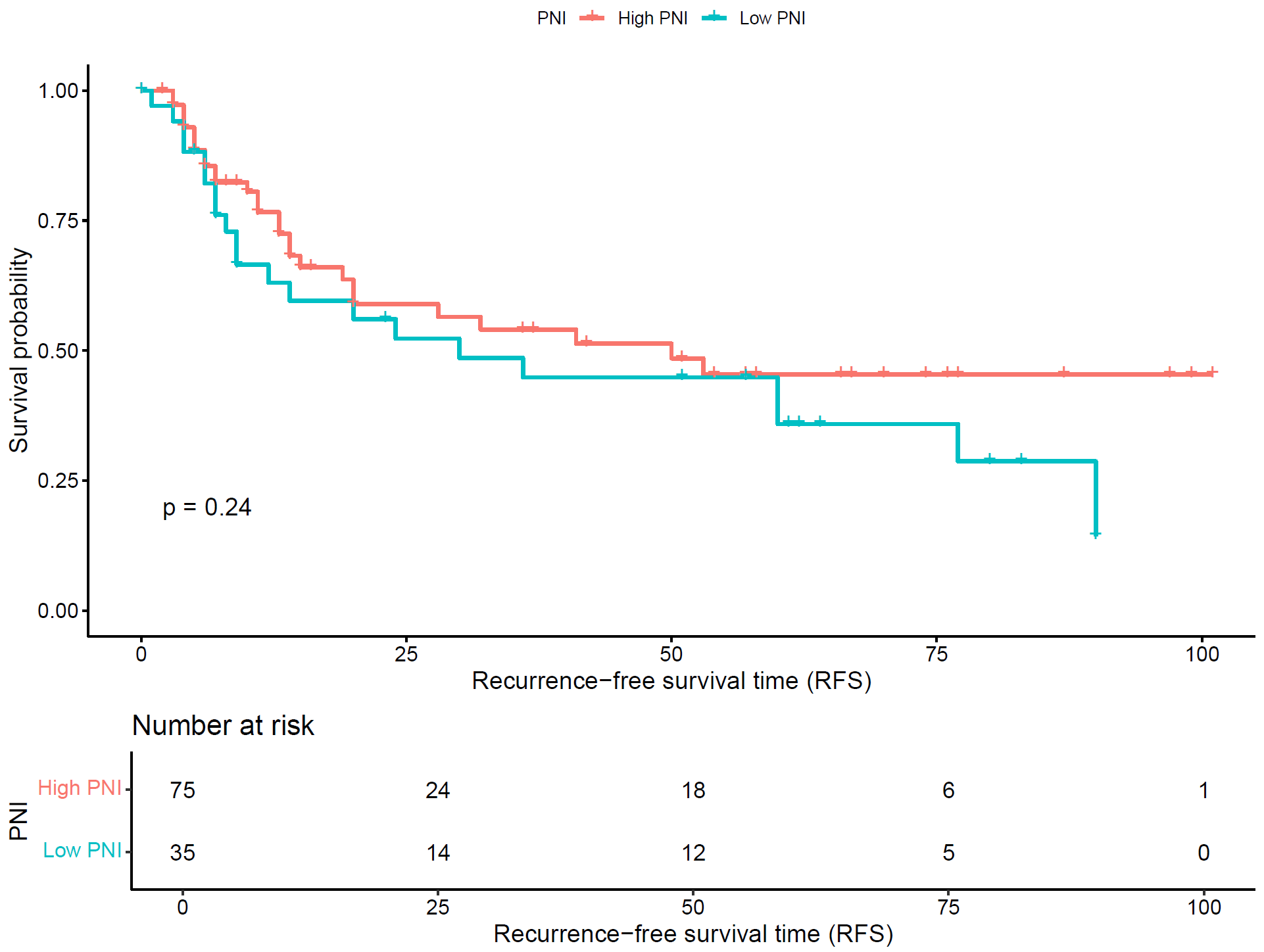
**

**SUPPLEMENTARY FIGURE S3(D).**

**
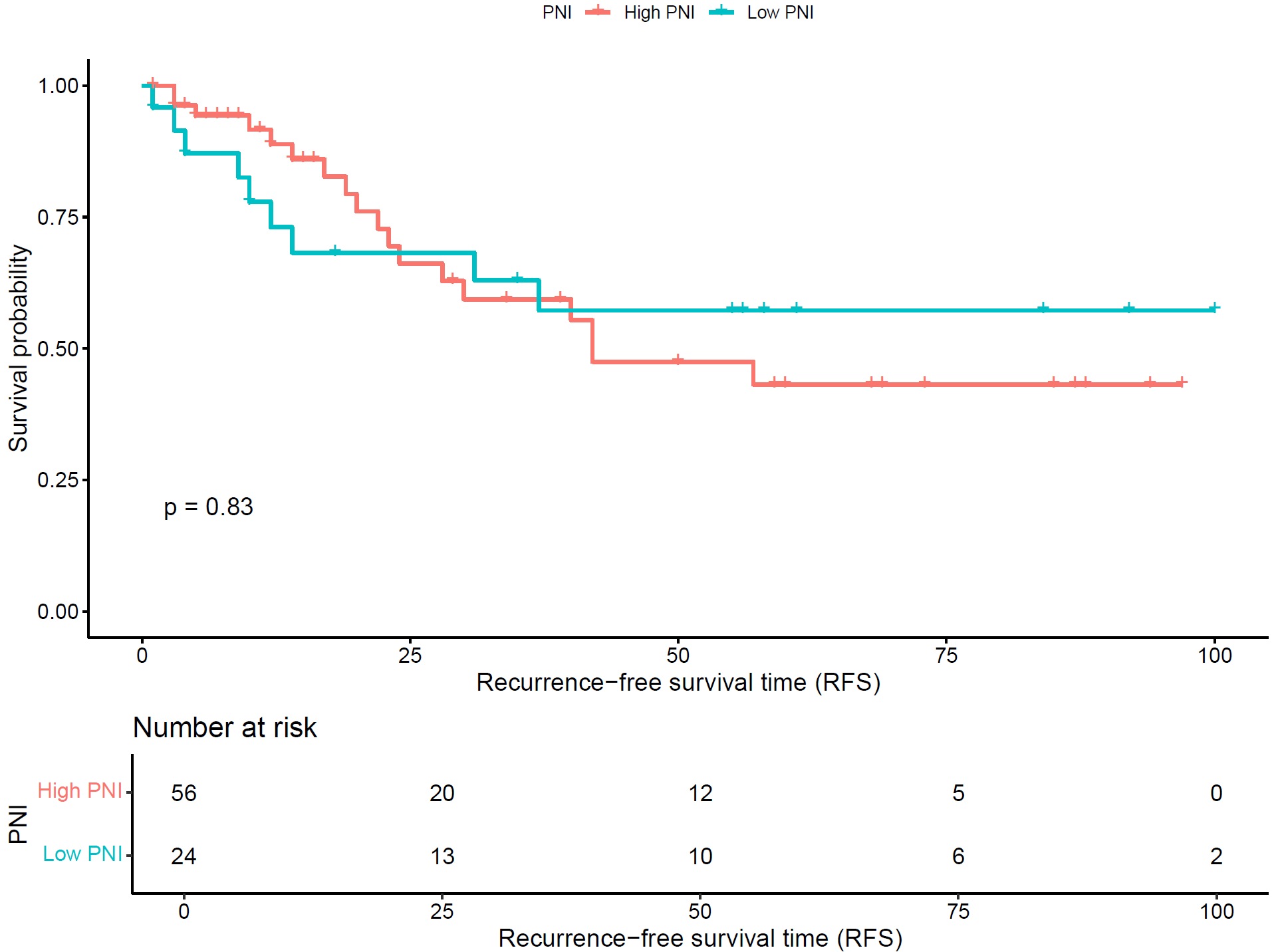
**

**SUPPLEMENTARY FIGURE S3(E).**

**
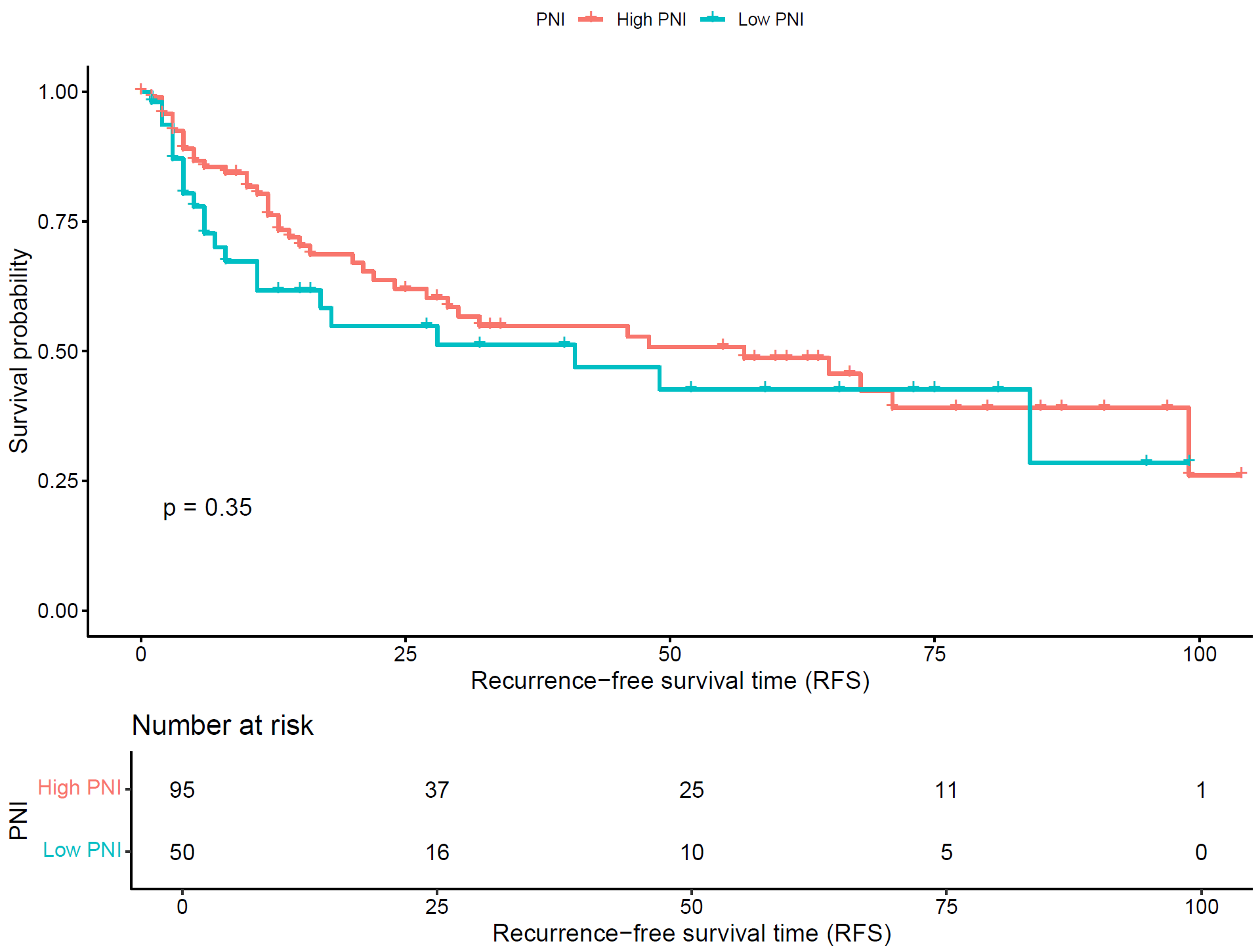
**

**SUPPLEMENTARY FIGURE S3(F).**

**
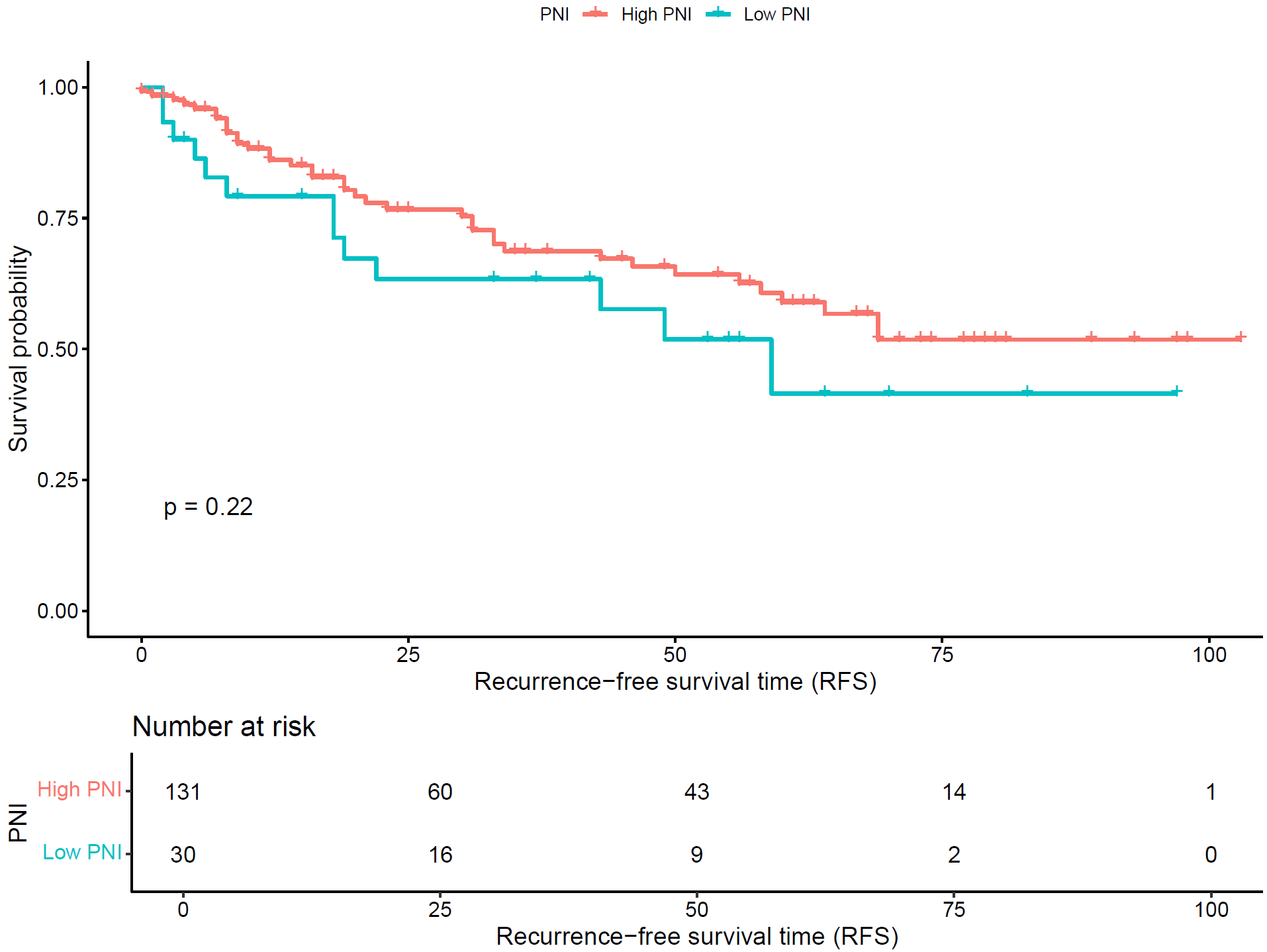
**

**SUPPLEMENTARY FIGURE S4(A)**

**
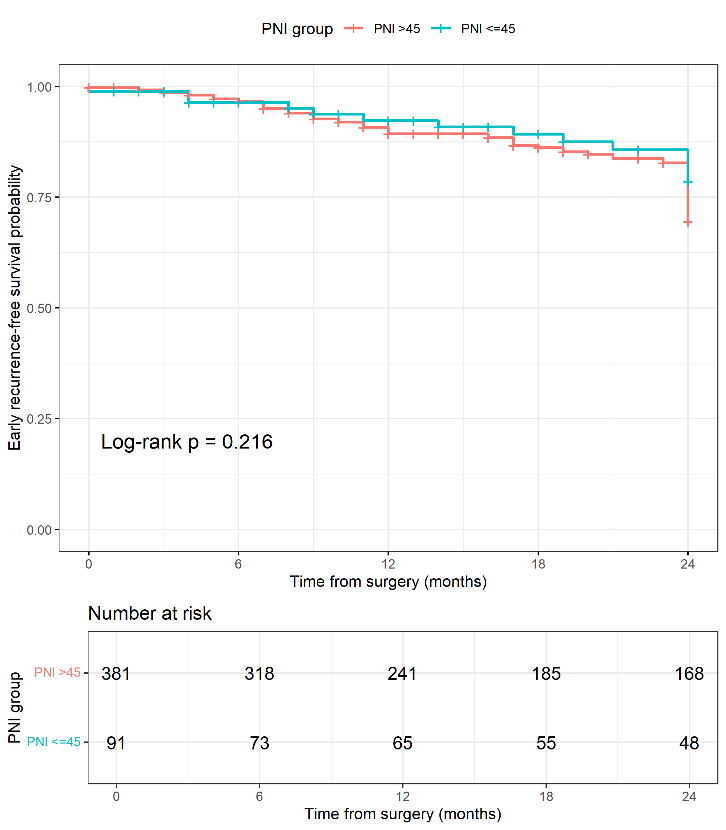
**

**SUPPLEMENTARY FIGURE S4(B)**

**
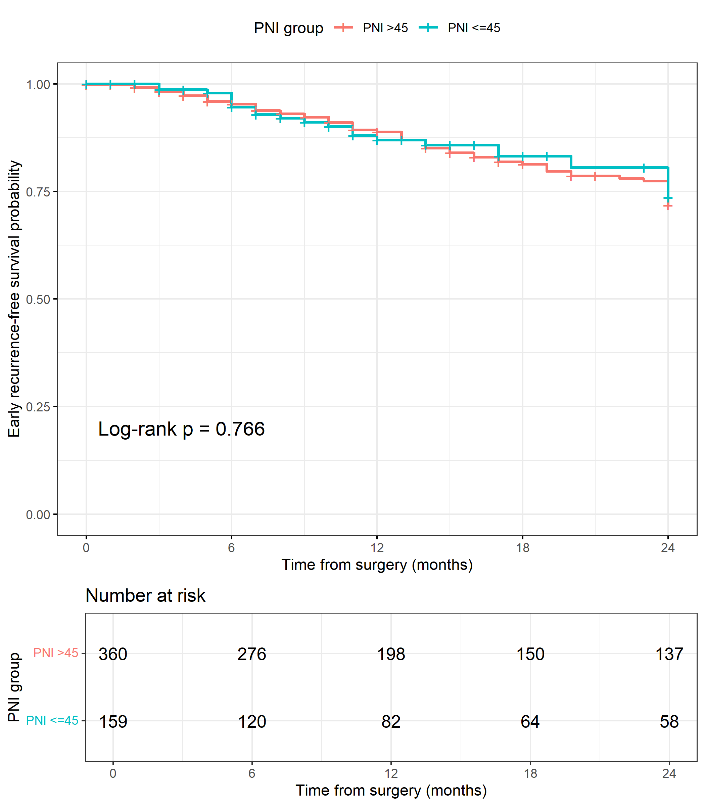
**

**SUPPLEMENTARY FIGURE S5(A)**

**
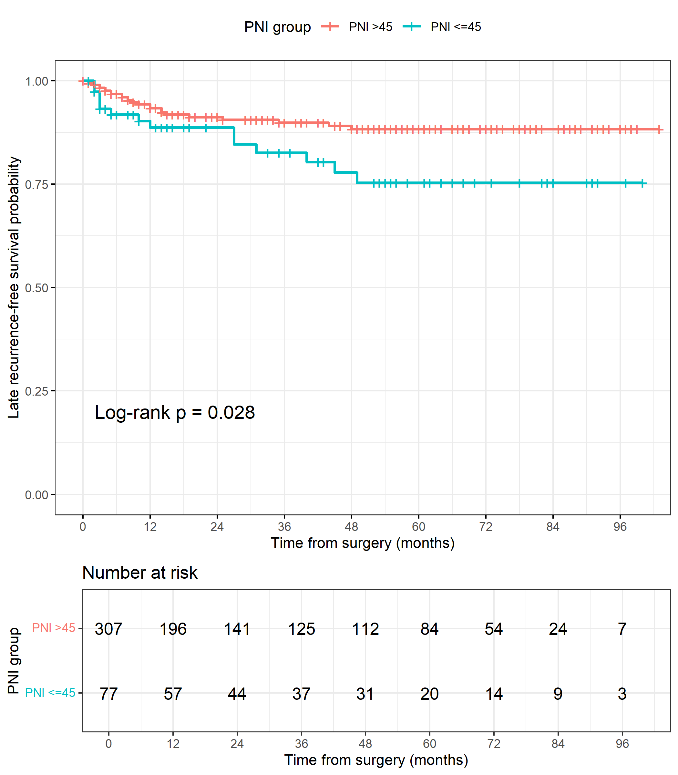
**

**SUPPLEMENTARY FIGURE S5(B).**

**
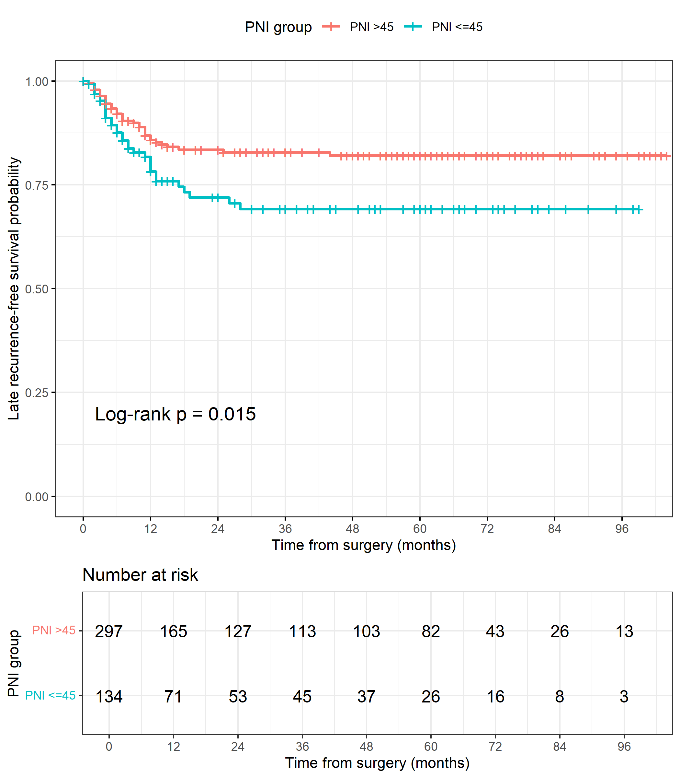
**

**SUPPLEMENTARY TABLE S1**

| **Parameter** | **Univariate**  **HR (95% CI)** | ***p* value** | **Multivariate**  **HR (95% CI)**  **Model 2: Alb** | ***p* value** |
| --- | --- | --- | --- | --- |
| **Age > 65** | 1.48 (1.12–1.95) | 0.005 |  |  |
| **Sex (F)** | 0.98 (0.72–1.33) | 0.881 |  |  |
| **HBsAg (+)** | 0.85 (0.65–1.11) | 0.236 |  |  |
| **Anti-HCV (+)** | 0.83 (0.57–1.19) | 0.306 |  |  |
| **Advanced stage** | 2.91 (2.20–3.83) | <0.001 | 1.75 (1.15-2.65) | 0.008 |
| **Multiple tumour** | 2.64 (2.01–3.47) | <0.001 |  |  |
| **Size > 5 cm** | 2.74 (2.08–3.60) | <0.001 |  |  |
| **Microscopic vessel invasion (+)** | 2.23 (1.66–3.00) | <0.001 |  |  |
| **High histology grade** | 1.62 (1.23–2.13) | <0.001 |  |  |
| **ISHAK grade > 4** | 0.97 (0.72–1.29) | 0.821 |  |  |
| **HAI score > 6** | 1.03 (0.78–1.35) | 0.850 |  |  |
| **Platelet < 100000** | 0.91 (0.68–1.21) | 0.503 |  |  |
| **INR > 1.1** | 1.34 (1.00–1.79) | 0.047 |  |  |
| **ALT > 40** (U/l) | 1.25 (0.94–1.65) | 0.123 |  |  |
| **AST > 45** (U/l) | 2.10 (1.59–2.77) | <0.001 | 1.38 (1.02-1.86) | 0.035 |
| **BILI > 1** (mg/dl) | 1.06 (0.78–1.44) | 0.721 |  |  |
| **Creatinine > 1.2** (mg/dl) | 1.68 (1.19–2.35) | 0.003 | 1.43 (1.01-2.04) | 0.046 |
| **AFP > 20** (ng/ml) | 1.89 (1.43–2.49) | <0.001 | 1.40 (1.04-1.89) | 0.028 |
| **ALBI grade 2 or 3** | 1.22 (0.85–1.75) | 0.288 |  |  |
| **FIB4 grade 2 or 3** | 1.63 (1.13–2.36) | 0.009 | 1.67 (1.13-2.47) | 0.010 |
| **ALB > 3.5** | 0.54 (0.30–0.79) | <0.002 |  |  |
| **BMI > 23** | 0.73 (0.55–0.97) | 0.027 |  |  |
| **Type 2 DM** | 0.98 (0.73–1.30) | 0.876 |  |  |
| **HTN** | 1.36 (1.03–1.78) | 0.030 |  |  |
| **Dyslipidaemia** | 0.98 (0.74–1.30) | 0.876 |  |  |
| **MASLD (+)** | 0.62 (0.47–0.82) | <0.001 |  |  |
| **MASH (+)** | 1.17 (0.77–1.80) | 0.459 |  |  |

**Abbreviations**: INR, International Normalized Ratio; ALT, Alanine Transaminase; AST, Aspartate Transaminase; AFP, Alpha-fetoprotein; ALB, albumin; ALBI, Albumin-Bilirubin; FIB-4, Fibrosis-4; BMI, Body Mass Index. MASLD, Metabolic Dysfunction-Associated Steatotic Liver Disease; MASH, Metabolic Dysfunction-Associated Steatohepatitis; HTN, Hypertension.

**SUPPLEMENTARY TABLE S2**

| **Parameter** | **Univariate  HR (95% CI)** | ***p* value** | **Multivariate  HR (95% CI)**  **Model 2: Alb** | ***p* value** |
| --- | --- | --- | --- | --- |
| **Age > 65** | 1.19 (0.98–1.46) | 0.085 |  |  |
| **Sex (F)** | 1.09 (0.87–1.36) | 0.442 |  |  |
| **HBsAg (+)** | 1.00 (0.82–1.22) | 0.994 |  |  |
| **Anti-HCV (+)** | 1.09 (0.85–1.40) | 0.501 |  |  |
| **Advanced stage** | 2.36 (1.93–2.89) | <0.001 | 1.86 (1.48-2.34) | <0.001 |
| **Multiple tumour** | 1.93 (1.58–2.37) | <0.001 |  |  |
| **Size > 5 cm** | 2.06 (1.69–2.52) | <0.001 |  |  |
| **Microscopic vessel invasion (+)** | 2.05 (1.66–2.53) | <0.001 | 1.45 (1.14-1.84) | 0.003 |
| **High histology grade** | 1.29 (1.05–1.59) | 0.014 |  |  |
| **ISHAK grade > 4** | 1.07 (0.87–1.32) | 0.540 |  |  |
| **HAI score > 6** | 1.11 (0.91–1.36) | 0.312 |  |  |
| **Platelet < 100000** | 0.96 (0.78–1.19) | 0.725 |  |  |
| **INR > 1.1** | 1.28 (1.03–1.59) | 0.025 | 1.26 (1.01-1.57) | 0.039 |
| **ALT > 40** (U/l) | 1.36 (1.11–1.67) | 0.004 |  |  |
| **AST > 45** (U/l) | 1.76 (1.42–2.17) | <0.001 | 1.33 (1.05-1.67) | 0.016 |
| **BILI > 1** (mg/dl) | 0.96 (0.76–1.21) | 0.725 |  |  |
| **Creatinine > 1.2** (mg/dl) | 1.14 (0.86–1.49) | 0.360 |  |  |
| **AFP > 20** (ng/ml) | 1.49 (1.22–1.82) | <0.001 | 1.28 (1.04-1.58) | 0.021 |
| **ALBI grade 2 or 3** | 1.14 (0.86–1.50) | 0.357 |  |  |
| **FIB4 grade 2 or 3** | 1.32 (1.02–1.71) | 0.034 |  |  |
| **ALB > 3.5** | 0.68 (0.50-0.93) | 0.016 |  |  |
| **BMI > 23** | 0.89 (0.72–1.09) | 0.264 |  |  |
| **Type 2 DM** | 1.01 (0.82–1.24) | 0.933 |  |  |
| **HTN** | 1.32 (1.08–1.61) | 0.007 | 1.33 (1.08-1.64) | 0.007 |
| **Dyslipidaemia** | 0.97 (0.79–1.20) | 0.803 |  |  |
| **MASLD (+)** | 0.76 (0.62–0.93) | 0.008 |  |  |
| **MASH (+)** | 1.05 (0.76–1.46) | 0.755 |  |  |

**Abbreviations**: INR, International Normalized Ratio; ALT, Alanine Transaminase; AST, Aspartate Transaminase; AFP, Alpha-fetoprotein; ALB, albumin; ALBI, Albumin-Bilirubin; FIB-4, Fibrosis-4; BMI, Body Mass Index. MASLD, Metabolic Dysfunction-Associated Steatotic Liver Disease; MASH, Metabolic Dysfunction-Associated Steatohepatitis; HTN, Hypertension.

**SUPPLEMENTARY TABLE S3**

| **Parameter** | **HR (95% CI)** | **p-value** |
| --- | --- | --- |
| **MASLD: OS** |  |  |
| Advanced stage | 2.14 (1.58–2.91) | <0.001 |
| FIB-4 grade 2 or 3 | 1.40 (1.13–1.74) | 0.002 |
| PNI > 50 | 0.64 (0.39-1.03) | 0.007 |
| **MASLD: RFS** |  |  |
| AFP > 20 (ng/ml) | 1.43 (1.05–1.96) | 0.024 |
| FIB-4 grade 2 or 3 | 1.43 (1.14–1.81) | 0.002 |
| Advanced stage | 2.21 (1.37–3.56) | 0.001 |

**Abbreviations**: AFP, Alpha-fetoprotein; FIB-4, Fibrosis-4.
